# Geographical Variation in Outcomes of Primary Hip and Knee Replacement

**DOI:** 10.1001/jamanetworkopen.2019.14325

**Published:** 2019-10-30

**Authors:** Cesar Garriga, José Leal, Maria T. Sánchez-Santos, Nigel Arden, Andrew Price, Daniel Prieto-Alhambra, Andrew Carr, Amar Rangan, Cyrus Cooper, George Peat, Raymond Fitzpatrick, Karen Barker, Andy Judge

**Affiliations:** 1 Nuffield Department of Orthopaedics, Rheumatology, and Musculoskeletal Sciences, Nuffield Orthopaedic Centre, University of Oxford, Oxford, United Kingdom; 2 Centre for Statistics in Medicine, Nuffield Department of Orthopaedics, Rheumatology, and Musculoskeletal Sciences, Nuffield Orthopaedic Centre, University of Oxford, Oxford, United Kingdom; 3 Nuffield Department of Population Health, University of Oxford, Oxford, United Kingdom; 4 MRC Lifecourse Epidemiology Unit, University of Southampton, Southampton General Hospital, Southampton, United Kingdom; 5 Department of Health Sciences, University of York, Heslington, York, United Kingdom; 6 National Joint Registry for England, Wales, Northern Ireland, and the Isle of Man, London, United Kingdom; 7 Research Institute for Primary Care and Health Sciences, Keele University, Keele, United Kingdom; 8 Physiotherapy Research Unit, Nuffield Orthopaedic Centre, Headington, Oxford, United Kingdom; 9 Musculoskeletal Research Unit, Translational Health Sciences, Bristol Medical School, University of Bristol, Southmead Hospital, Bristol, United Kingdom

## Abstract

**Importance:**

Little is known about variation in outcomes of surgery or about the factors associated with such variation.

**Objectives:**

To evaluate variation in patient outcomes and costs for primary hip and knee replacement across health areas in England and to identify whether patient, surgical, or hospital factors are associated with such variation.

**Design, Setting, and Participants:**

This cohort study used data from the National Joint Registry, linked to English Hospital Episode Statistics and Patient Reported Outcome Measures data sets, for 383 382 adult patients who underwent primary total hip replacement (THR) or primary total and unicompartmental knee replacement (TKR) surgical procedures from January 2014 to December 2016. Geographical Information Systems were used to display maps describing adjusted estimates of variation in outcomes across health areas. Data analysis took place from January 2018 to August 2019.

**Exposures:**

Patient characteristics (eg, age, sex, body mass index [BMI], and socioeconomic deprivation), surgical factors (eg, surgeon volume and grade), and hospital organizational factors (eg, number of operating theaters, number of specialist consultants, and hospital volume).

**Main Outcomes and Measures:**

Length of stay (LOS), bed-day costs, change in Oxford hip or knee scores 6 months after surgery, and complications 6 months after surgery.

**Results:**

A total of 173 107 patients (mean [SD] age, 69.3 [10.7] years; mean [SD] BMI, 28.9 [5.2]) underwent primary THR and 210 275 patients (mean [SD] age 69.7 [9.4] years; mean [SD] BMI, 31.1 [5.5]) underwent primary TKR, nested in 207 health areas. A number of factors were associated with longer LOS, higher bed-day costs, smaller changes in Oxford hip or knee scores, and a higher percentage of complications, including a workforce with a higher number of less experienced physicians (eg, LOS for less experienced surgeons, THR: regression coefficient, 0.02; 95% CI, 0.01 to 0.03; *P* < .001; TKR: regression coefficient, 0.01; 95% CI, 0.01 to 0.02; *P* < .001), public hospitals (eg, bed-day costs for private hospitals, THR: regression coefficient, −0.15; 95% CI, −0.15 to −0.14; *P* < .001; TKR: regression coefficient, −0.19; 95% CI, −0.19 to −0.19; *P* < .001), low volume of surgical procedures per surgeon (eg, change in Oxford hip or knee scores for lead surgeon with ≤10 vs >150 surgical procedures per year, THR: regression coefficient, −1.03; 95% CI, −1.47 to −0.58; *P* < .001; TKR: regression coefficient, −0.54; 95% CI, −1.01 to −0.06), and low volume of surgical procedures per hospital (eg, percentage of complications for hospitals with ≤200 vs ≥500 surgical procedures per year, THR: regression coefficient, 0.12; 95% CI, 0.04 to 0.21; *P* < .001; TKR: regression coefficient, 0.09; 95% CI, 0.01 to 0.18; *P* = .03). Although these factors did not attenuate the magnitude of variation across health areas, they had ecological correlations with the observed geographical variations in outcomes of surgery by health area. For example, the percentage of public and private hospitals was ecologically correlated at the health area level with longer and shorter stays, respectively (public hospital, THR: ρ, 0.41; public hospital, TKR: ρ, 0.44; private hospital, THR: ρ, −0.37; private hospital, THR: ρ, −0.38). Across health areas, estimated mean length of stay ranged from 3 to 7 days, and associated bed-day costs ranged from £4727 ($5827) to £8800 ($10 848) for both total hip and knee replacement. The absolute estimated mean change in Oxford hip score varied from 18.7 to 24.6 points and, for Oxford knee score, from 13.1 to 18.8. Estimated 6-month complications ranged from 2.9% to 5.8% for both THR and TKR.

**Conclusions and Relevance:**

In this study, models indicated that higher surgical volume by surgeon and by hospital as well as private hospitals were associated with better patient outcomes, which could be explained by the changing case mix of public hospitals treating an increasing number of more complex patients. A higher proportion of less experienced physicians was associated with poorer outcomes. This variation was observed geographically.

## Key Points

**Question:**

Are hospital organizational factors, surgical factors, and patient factors associated with patient outcomes and National Health Service costs for total hip and knee replacement?

**Findings:**

This cohort study of more than 383 000 patients in 207 health areas in England identified significant health area–level variation in patient outcomes for total hip and knee replacement surgery. Geographical variation was associated with better outcomes for surgical procedures in private and high-volume hospitals as well as for operations performed by more experienced surgeons with a higher volume of operations per year.

**Meaning:**

Findings are informative for commissioners in monitoring variations in surgical outcomes and for patients deciding where to undergo surgery.

## Teaser

This cohort study evaluates variation in patient outcomes and costs for primary hip and knee replacement across health areas in England and identifies whether patient, surgical, or hospital factors are associated with such variation.

## Introduction

Commissioners of health care, who are responsible for health services, need to be concerned about the quality of health care that they commission, with a focus on quality improvement and reducing unwarranted variations in quality and outcome.^1^ In the United Kingdom, the National Health Service (NHS) Act 2006, as amended by the Health and Social Care Act 2012, places duties on the NHS Commissioning Board and local clinical commissioning groups (CCGs) to work to reduce variations in access to and outcomes from health care services for patients. These commissioners are also required to assess and report on how well they have fulfilled this duty.^2^

There are well-known geographical variations in the provision of common surgical procedures, including total hip replacement (THR) and total or unicompartmental knee replacement (TKR),^3^ as publicized through the NHS Atlas of Variation.^1^ For example, adjusted rates of provision per 1000 individuals in need of hip replacement ranged between a rate ratio of 12.2 and 144.0 across English health districts.^4^ A 2014 study^5^ found evidence of significant unexplained variation between hospitals in health outcomes and resource use following THR and TKR, but little is known about the factors associated with such variation. We hypothesized that outcomes of surgery will vary across different hospitals and areas of the country,^5^ that these variations may be associated with differences in case mix, eg, more complex cases and patients with poorer health, and that this phenomenon must be accounted for. However, differences in patient outcomes could also be associated with how hospitals organize their services,^4^ eg, bed availability, numbers of operating theaters and specialist surgeons, the use of new surgical techniques, such as minimally invasive surgery,^6^ and centralizing care into specialist high-volume hospitals.^7^ Knowledge of these factors would help to change the way services are organized, improve the quality of health care, and reduce geographical variation in patient outcomes across health areas.

The specific gaps in knowledge that this article aims to address are as follows: (1) describe geographical variation in patient outcomes for THR and TKR across different commissioning health areas of England and (2) explore whether patient case mix, surgical, and/or hospital organizational factors are associated with such variation.

## Methods

### Study Design and Data Source

We performed a retrospective cohort study using data obtained from the National Joint Registry (NJR), which contains data on 2 million THR and TKR replacement operations. Before personal data and sensitive personal data are recorded, express written patient consent is provided. With support under Section 251 of the NHS Act 2006, the ethics and confidentiality committee allows the NJR to collect patient data where consent is indicated as not recorded. The ethical approval granted to NJR also applied to this study. Primary operations were linked with Hospital Episode Statistics (HES) data, which contain records of all inpatient episodes undertaken in NHS trusts in England (125 million per year). In turn, primary THR and TKR were linked to Patient Reported Outcome Measures. Patients funded by the NHS in England are asked to complete questionnaires to evaluate their perception of improvement in health. Hospital organizational factors (ie, workforce, bed availability, and operating theaters) were retrieved and linked to HES from the Hospital and Community Health Service Workforce Statistics, the Quarterly Bed Availability and Occupancy, and the Supporting Facilities data sets. Two cohorts who underwent primary THR and TKR operations were retrieved for January 2014 through December 2016, in England. We followed the Strengthening the Reporting of Observational Studies in Epidemiology (STROBE) reporting guideline.

### Main Outcome Measures

We evaluated hospital length of stay (LOS) for patients undergoing primary THR and TKR. Length of stay was calculated as the number of days between the hospital admission date and the hospital discharge date. For the same set of patients used to estimate LOS, we estimated the inpatient cost associated with the index episode, using NHS reference costs from 2015 to 2016.^8^ We estimated the mean cost per bed-day based on health care resource group, which refers to standard groupings of clinically similar treatments that use common levels of health care resources, for each patient and their LOS (eAppendix 1 in the Supplement).

Additionally, we assessed absolute change in Oxford hip and knee scores (OHS and OKS, respectively). Patients completed a questionnaire about their pain and function before surgery and 6 months after the surgery to measure early functional recovery.^9^ A total score is calculated from 0 to 48, where 0 is the worst possible score (most severe symptoms) and 48 is the best (fewest symptoms). We calculated the difference between the total scores 6 months after the operation and at baseline to obtain a measure of change associated with the surgery. A higher positive value for OHS or OKS change represented greater improvement in pain and function. We defined postoperative complications as 1 or more events that occurred up to 6 months after the primary replacement operation that would likely be related to the surgery (eAppendix 2 and eAppendix 3 in the Supplement).

### Potential Factors

We classified potential factors as patient, surgical, or hospital organizational factors (eTable 1 in the Supplement). Patient factors included calendar year of THR or TKR; age; sex; body mass index, calculated as weight in kilograms divided by height in meters squared^10^; American Society of Anesthesiologists (ASA) grade^11^; area-level socioeconomic deprivation using the Index of Multiple Deprivation, based on patient residential post code; rural vs urban indicator; primary indication; Charlson Comorbidity Index (CCI) score; baseline OHS or OKS score; and the baseline European Quality of Life-Five Domain (EQ-5D-3L) score. Surgical factors included lead surgeon experience, surgical volume per lead surgeon and year, surgical volume per unit and year, minimally invasive surgery (yes or no), thromboprophylaxis, mechanical prophylaxis, anesthetic type, and type of approach. Hip bone grafts were classified as femoral bone graft or cup bone graft. Knee bone grafts were classified as femoral bone graft or tibial bone graft. Hip implant fixation categories included cup fixation and stem fixation, except resurfacing. Knee implant fixation categories included femoral fixation and tibial fixation. We also included the type of hip implant by bearing surface and by femoral head size as well as the type of knee implant. Hospital organizational factors included unit type, full-time equivalent (FTE; ie, proportion of full-time contracted hours) of specialty groups on trauma and orthopedic surgery, FTE consultants, FTE midgrade physicians, FTE early-career physicians, total beds available overnight, total beds available overnight for trauma and orthopedic surgery, total beds available overnight for rehabilitation, number of operating theaters, and number of dedicated day-case operating theaters.

### Exclusion Criteria

We included only patients receiving elective surgery (eFigure 1 in the Supplement). We excluded patients without information about the census lower level super output area used to group patients in geographical areas, which is necessary to conduct the multilevel modeling. Patients with missing data for LOS were also excluded. We excluded patients without information on baseline or 6-month OHS or OKS scores for the analysis of change.

### Missing Data

We used the Pearson χ^2^ statistic to evaluate missingness for OHS and OKS across categories of confounders listed earlier. We compared the distribution of patients with and without data for OHS and OKS by categories of those confounders. We generated a single imputed data set using a chained equation.

### Patient and Public Involvement

Among the priorities identified through the work of the James Lind Alliance Priority Setting Partnership for Hip/Knee Replacement was the need to involve patients in identifying the outcomes that matter most to them.^12^ We used the University of Bristol Musculoskeletal Research Unit patient involvement group, the Patient Experience Partnership in Research.^13^ This group comprises 12 patients with musculoskeletal conditions. The outcomes most important to the group were pain and function. Complications were considered important, particularly infection. The group agreed that LOS was an important outcome but very dependent on the level of support at home. Revision, reoperation, and mortality were ranked low by the group.

### Statistical Analysis

The hierarchical structure of the data consisted of patients (level 1), nested in lower level super output area (level 2) and in CCGs (level 3). Multilevel regression models were used to describe the association of patient, surgical, and hospital organization factors on patient outcomes of surgery. This controlled for evidence of clustering in the data by allowing outcomes to vary across lower level super output area and CCG. Failure to control for evidence of clustering can lead to estimates of standard errors that are spuriously precise and be a potential source of bias. Analyses were conducted separately for THR and TKR. We excluded nonsignificant terms using a backward approach to maximize statistical power, producing models with meaningful selected variables. The overall outcome was estimated for each CCG. We fitted the following models: (1) null model of actual observed outcomes, (2) model adjusted for patient case-mix variables, (3) model further adjusted for surgical variables, and (4) model further adjusted for hospital organizational variables. We produced ecological correlations of hospital factors at the health area–level with estimated outcomes, using Pearson correlation tests. Variation in outcomes was presented using maps of the 2017 CCG areas.

Analyses were conducted using Stata version 15.1 (StataCorp), MLwiN version 3.00 (Centre for Multilevel Modeling), and R version 3.5.0 (R Project for Statistical Computing). Statistical significance was set at *P* = .05, and all tests were 2-tailed.

## Results

Between 2014 and 2016, there were 173 107 primary THRs and 210 275 primary TKRs (eFigure 1 in the Supplement). A total of 223 296 surgical procedures (58.3%) were performed on women. Patients undergoing THR had a mean (SD) age of 69.3 (10.7) and a mean (SD) body mass index of 28.9 (5.2); patients undergoing TKR had a mean (SD) age of 69.7 (9.4) and mean (SD) body mass index of 31.1 (5.5) (Table 1). The American Society of Anesthesiologists grade of patients was mild (ie, 2) or fit (ie, 1) for patients in 317 452 surgical procedures (82.8%). Additional patient, surgical, and hospital organization factors are summarized for THR and TKR in Table 1 and Table 2.

**Table 1.** Distribution of Patient and Surgical Factors Associated With Patient Outcomes of Surgery

Factor	No. (%)	
	THR (n = 173 107)	TKR or UKR (n = 210 725)
**Patient Factors**		
Calendar year		
2014	57 156 (33.0)	69 001 (32.7)
2015	57 535 (33.2)	69 999 (33.2)
2016	58 416 (33.8)	71 725 (34.0)
Age, y		
<50	7907 (4.6)	4180 (2.0)
50-59	22 887 (13.2)	26 789 (12.7)
60-69	51 097 (29.5)	68 970 (32.7)
70-79	61 994 (35.8)	79 241 (37.6)
80-84	19 426 (11.2)	21 753 (10.3)
≥85	9796 (5.7)	9792 (4.7)
Women	103 860 (60.0)	119 436 (56.7)
BMI, mean (SD)	28.9 (5.2)	31.1 (5.5)
Preoperative ASA physical function score		
1, Fit and healthy	22 195 (12.8)	18 909 (9.0)
2, Mild disease, not incapacitating	121 255 (70.1)	155 093 (73.6)
3, Incapacitating systemic disease	28 945 (16.7)	36 171 (17.2)
4-5, Life-threatening disease or expected to die within 24 h	712 (0.4)	552 (0.3)
IMD quintile		
Lowest quintile, least deprived	42 058 (24.3)	46 435 (22.0)
Second quintile	43 001 (24.8)	49 019 (23.3)
Third quintile	31 200 (18.0)	40 216 (19.1)
Fourth quintile	29 289 (16.9)	38 292 (18.2)
Highest quintile, most deprived	27 559 (15.9)	36 763 (17.5)
Rural/urban indicator		
Urban	123 862 (71.6)	157 758 (74.9)
Town and fringe	22 223 (12.8)	25 083 (11.9)
Village or isolated	27 022 (15.6)	27 884 (13.2)
Primary indication		
Osteoarthritis	167 686 (96.9)	208 333 (98.9)
Osteoarthritis and other^a^	5421 (3.1)	2392 (1.1)
Charlson Comorbidity Index score		
0	122 047 (70.5)	140 711 (66.8)
1	32 926 (19.0)	46 984 (22.3)
2	11 870 (6.9)	15 341 (7.3)
≥3	6264 (3.6)	7689 (3.7)
Baseline OHS or OKS, median (IQR)	17 (11-23)	18 (12-23)
EQ-5D-3L quintile		
Lowest quintile, lowest quality of life	21 265 (12.3)	40 798 (19.4)
Second quintile	47 303 (27.3)	29 601 (14.1)
Third quintile	35 237 (20.4)	54 583 (25.9)
Fourth quintile	32 083 (18.5)	20 352 (9.7)
Highest quintile, highest quality of life	37 219 (21.5)	65 391 (31.0)
**Surgical Factors**		
Lead surgeon experience		
Consultant	143 417 (82.9)	172 183 (81.7)
Other^b^	29 690 (17.2)	38 542 (18.3)
Surgical procedures per lead surgeon, No./y		
≤10	3013 (1.4)	3109 (1.8)
11-50	45 279 (21.5)	37 816 (21.9)
51-75	41 610 (19.8)	30 651 (17.7)
76-100	38 898 (18.5)	30 979 (17.9)
101-150	45 056 (21.4)	36 610 (21.2)
>150	36 869 (17.5)	33 942 (19.6)
Surgical procedures per unit, No./y		
≤200	45 319 (26.2)	31 321 (14.9)
200-299	47 040 (27.2)	51 335 (24.4)
300-399	37 984 (21.9)	50 346 (23.9)
400-499	10 159 (5.9)	32 782 (15.6)
≥500	32 605 (18.8)	44 941 (21.3)
Minimally invasive surgery	7076 (4.1)	9332 (4.4)
Thromboprophylaxis		
None	1598 (0.9)	2049 (1.0)
Aspirin only	5219 (3.0)	7111 (3.4)
LMWH, with or without other	109 443 (63.2)	152 836 (72.5)
Other, no LMWH	56 847 (32.8)	48 729 (23.1)
Mechanical prophylaxis	167 638 (96.8)	204 642 (97.1)
Anesthetic type		
General	56 951 (32.9)	62 447 (29.6)
Regional, epidural	5230 (3.0)	6841 (3.3)
Regional, nerve block	8824 (5.1)	21 619 (10.3)
Regional, spinal, intrathecal	131 627 (76.0)	157 123 (74.6)
Approach		
Anterior, antero-lateral, hardinge, lateral, trochanteric osteotomy, or other	55 100 (31.8)	NA
Posterior	118 007 (68.2)	NA
Lateral parapatellar	NA	1907 (0.9)
Medial parapatellar	NA	197 718 (93.8)
Midvastus	NA	5942 (2.8)
Subvastus	NA	2302 (1.1)
Other approaches in knee surgery	NA	2856 (1.4)
Bone grafts		
Femoral^c^	921 (0.5)	2225 (1.1)
Cup	5669 (3.3)	NA
Tibia	NA	705 (0.3)
Primary cup fixation		
Cementless	110 862 (64.4)	NA
Cemented	61 415 (35.7)	NA
Type of primary stem fixation		
Cementless	72 509 (42.4)	NA
Cemented	98 607 (57.6)	NA
Primary femoral fixation		
Cementless	NA	9461 (4.5)
Cemented	NA	200 741 (95.5)
Tibial fixation		
Cementless	NA	8855 (4.2)
Cemented	NA	201 226 (95.8)
Bearing surface		
MoM	991 (0.6)	NA
MoP	106 548 (62.1)	NA
CoC	20 821 (12.1)	NA
CoP	43 138 (25.2)	NA
CoM, MoC, or unknown	23 (0.1)	NA
Femoral head size, mm		
≤28	55 652 (32.4)	NA
32	75 536 (44.0)	NA
36-42	39 556 (23.0)	NA
≥44	1141 (0.7)	NA
Type of knee implant		
Total knee replacement	NA	194 464 (92.3)
Unicompartmental knee replacement	NA	16 261 (7.7)

Abbreviations: ASA, American Society of Anesthesiologists; BMI, body mass index (calculated as weight in kilograms divided by height in meters squared); CoC, ceramic-on-ceramic; CoM, ceramic-on-metal; CoP, ceramic-on-polyethylene; EQ-5D-3L, European Quality of Life-5 Domain; IMD, Index of Multiple Deprivation; IQR, interquartile range; LMWH, low-molecular-weight heparin; MoM, metal-on-metal; MoP, metal-on-polyethylene; MoC, metal-on-ceramic; NA, not applicable; OHS, Oxford hip score; OKS, Oxford knee score; THR, total hip replacement; TKR, total knee replacement; UKR, unicompartmental knee replacement.

^a^ List of indications appears in eTable 1 in the Supplement.

^b^ Other types of physicians appear in eTable 1 in the Supplement.

^c^ Implanted in head of the femur for hips and distal part of the femur for knees.

**Table 2.** Distribution of Hospital Factors Associated With Patient Outcomes of Surgery

Factor	No. (%)	
	THR (n = 173 107)	TKR or UKR (n = 210 725)
Unit type		
Public hospital	123 481 (71.3)	148 758 (70.6)
Private hospital	40 842 (23.6)	50 739 (24.1)
Private treatment center	8784 (5.1)	11 228 (5.3)
FTE specialty groups on trauma and orthopedic surgery, No.		
0-24	20 558 (11.9)	63 415 (30.1)
25-29	17 541 (10.1)	29 416 (14.0)
30-39	32 725 (18.9)	47 334 (22.5)
40-49	32 528 (18.8)	28 572 (13.6)
>50	69 755 (40.3)	41 988 (19.9)
FTE consultants, No.		
0-24	40 108 (23.2)	92 154 (43.7)
25-29	15 788 (9.1)	21 437 (10.2)
30-39	32 530 (18.8)	33 396 (15.9)
40-49	23 966 (13.8)	25 209 (12.0)
>50	60 715 (35.1)	38 529 (18.3)
FTE midgrade physicians, No.		
0-24	97 397 (56.3)	151 270 (71.8)
25-29	18 900 (10.9)	17 590 (8.4)
30-39	21 667 (12.5)	19 425 (9.2)
40-49	14 362 (8.3)	9306 (4.4)
>50	20 781 (12.0)	13 134 (6.2)
FTE early-career physicians, No.		
0-24	168 646 (97.4)	208 405 (98.9)
25-29	3757 (2.2)	2055 (1.0)
30-39	704 (0.4)	265 (0.1)
40-49	0	0
>50	0	0
Total beds available overnight, No.		
0-349	15 186 (8.8)	41 447 (19.7)
350-499	18 469 (10.7)	40 887 (19.4)
500-699	34 541 (20.0)	51 403 (24.4)
700-999	51 891 (30.0)	41 415 (19.7)
≥1000	53 020 (30.6)	35 573 (16.9)
Beds available overnight for trauma and orthopedic surgery, No.		
0-34	15 873 (9.2)	59 732 (28.4)
35-49	26 118 (15.1)	46 946 (22.3)
50-69	47 209 (27.3)	53 577 (25.4)
70-99	52 178 (30.1)	32 574 (15.5)
≥100	31 729 (18.3)	17 896 (8.5)
Beds available overnight for rehabilitation, No.		
0	74 465 (57.8)	59 793 (54.5)
>0-10	9605 (7.5)	8695 (7.9)
11-20	16 923 (13.1)	15 651 (14.3)
≥20	27 754 (21.6)	25 655 (23.4)
Operating theaters, No.		
<10	11 278 (6.5)	53 927 (25.6)
10-14	26 830 (15.5)	45 205 (21.5)
15-19	38 320 (22.1)	42 661 (20.2)
20-24	27 414 (15.8)	22 226 (10.6)
≥25	69 265 (40.0)	46 706 (22.2)
Dedicated day-case operating theaters, No.		
0	24 951 (14.4)	39 251 (18.6)
1-2	32 531 (18.8)	51 685 (24.5)
3-4	37 891 (21.9)	47 745 (22.7)
5-6	42 126 (24.3)	27 857 (13.2)
≥7	35 608 (20.6)	44 187 (21.0)

Abbreviation: FTE, full-time equivalent.

### Outcomes

#### LOS and Bed-Day Costs

**Figure 1.**
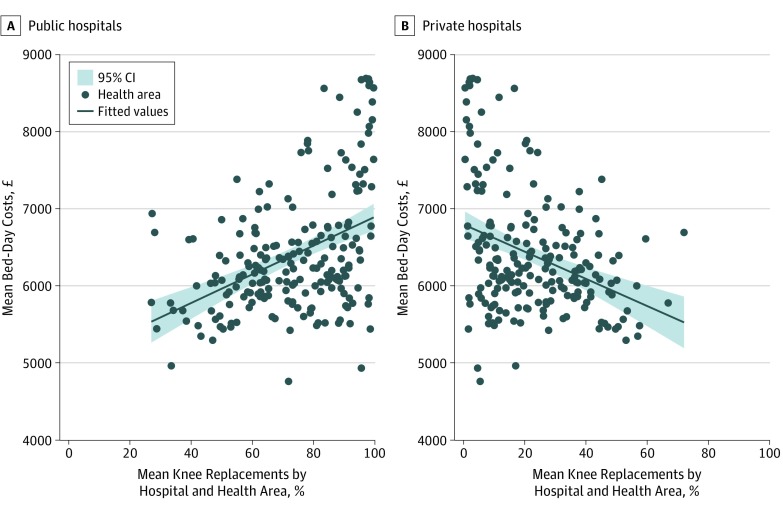
Correlation Between Bed-Day Costs and Percentage of Knee Replacements in Public and Private Hospitals by Health Area in England, 2014-2016 To convert pounds sterling to US dollars, multiply by 1.23.

Longer LOS was associated with patients aged 80 years or older (80-84 years, THR: regression coefficient, 0.39; 95% CI, 0.38 to 0.41; *P* < .001; TKR: regression coefficient, 0.32; 95% CI, 0.30 to 0.34; *P* < .001; ≥85 years, THR: regression coefficient, 0.59; 95% CI, 0.57 to 0.61; *P* < .001; TKR: regression coefficient, 0.49; 95% CI, 0.47 to 0.51; *P* < .001), those with an ASA grade of 3 or higher (grade 3, THR: regression coefficient, 0.17; 95% CI, 0.17 to 0.18; *P* < .001; TKR: regression coefficient, 0.15; 95% CI, 0.15 to 0.16; *P* < .001; grade 4-5, THR: regression coefficient, 0.36; 95% CI, 0.33 to 0.39; *P* < .001; TKR: regression coefficient, 0.32; 95% CI, 0.29 to 0.35; *P* < .001), and those with a CCI score of 2 or higher (2, THR: regression coefficient, 0.16; 95% CI, 0.15 to 0.17; *P* < .001; TKR: regression coefficient, 0.16; 95% CI, 0.15 to 0.16; *P* < .001; ≥3, THR: regression coefficient, 0.30; 95% CI, 0.29 to 0.32; *P* < .001; TKR: regression coefficient, 0.28; 95% CI, 0.27 to 0.29; *P* < .001). Shorter LOS was associated with private hospitals (THR: regression coefficient, −0.22; 95% CI, −0.23 to −0.21; *P* < .001; TKR: regression coefficient, −0.26; 95% CI, −0.27 to −0.26; *P* < .001) or private treatment centers (THR: regression coefficient, −0.40; 95% CI, −0.41 to −0.38; *P* < .001; TKR: regression coefficient, −0.44; 95% CI, −0.45 to −0.42; *P* < .001), high-volume hospitals (≤200 vs ≥500 surgical procedures per year, THR: regression coefficient, 0.14; 95% CI, 0.13 to 0.15; *P* < .001; TKR: regression coefficient, 0.10; 95% CI, 0.09 to 0.11; *P* < .001), operations performed by lead surgeons (less-experienced surgeons, THR: regression coefficient, 0.02; 95% CI, 0.01 to 0.03; *P* < .001; TKR: regression coefficient, 0.01; 95% CI, 0.01 to 0.02; *P* < .001), and among patients in the highest quintile of EQ-5D-3L scores (fourth quintile vs highest quintile, THR: regression coefficient, −0.22; 95% CI, −0.23 to −0.21; *P* < .001; highest quintile vs lowest quintile, TKR: regression coefficient, −0.18; 95% CI, −0.19 to −0.18; *P* < .001) (eTable 2 and eTable 3 in the Supplement). Hospitals with 100 or more beds available overnight for trauma and orthopedics were associated with longer LOS for THR than hospitals with fewer than 35 beds (regression coefficient, 0.16; 95% CI, 0.14 to 0.17; *P* < .001) (eTable 2 in the Supplement). Patients undergoing TKR were associated with longer LOS than those undergoing UKR (regression coefficient, 0.28; 95% CI, 0.27 to 0.29; *P* < .001) (eTable 3 in the Supplement). The percentage of public and private hospitals was ecologically correlated at CCG level with longer and shorter stays, respectively (public hospital, THR: ρ, 0.41; public hospital, TKR: ρ, 0.44; private hospital, THR: ρ, −0.37; private hospital, THR: ρ, −0.38) (Figure 1; eTable 4 in the Supplement).

Observed mean bed-day costs by CCG ranged between £4322 ($5328) and £8566 ($10 559) for THR and £4564 ($5626) to £8901 ($10 972) for TKR. Higher bed-day costs were found for older patients (≥85 years, THR: regression coefficient, 0.42; 95% CI, 0.42 to 0.42; *P* < .001; TKR: regression coefficient, 0.35; 95% CI, 0.35 to 0.35; *P* < .001), those with an ASA grade of 3 or higher (grade 3, THR: regression coefficient, 0.09; 95% CI, 0.09 to 0.09; *P* < .001; TKR: regression coefficient, 0.09; 95% CI, 0.09 to 0.09; *P* < .001; grade 4-5, THR: regression coefficient, 0.18; 95% CI, 0.18 to 0.18; *P* < .001; TKR: regression coefficient, 0.17; 95% CI, 0.17 to 0.17; *P* < .001), and those with a CCI score of 3 or higher (THR: regression coefficient, 0.08; 95% CI, 0.08 to 0.08; *P* < .001; TKR: regression coefficient, 0.09; 95% CI, 0.09 to 0.09; *P* < .001) (eTable 5 and eTable 6 in the Supplement). Lower bed-day costs were associated with private hospitals (THR: regression coefficient, −0.15; 95% CI, −0.15 to −0.14; *P* < .001; TKR: regression coefficient, −0.19; 95% CI, −0.19 to −0.19; *P* < .001) or private treatment centers (THR: regression coefficient, −0.29; 95% CI, −0.29 to −0.29; *P* < .001; TKR: regression coefficient, −0.33; 95% CI, −0.33 to −0.33; *P* < .001), high-volume lead surgeons and hospitals (lead surgeon with ≤10 vs >150 surgical procedures per year, THR: regression coefficient, 0.02; 95% CI, 0.02 to 0.02; *P* < .001; TKR: regression coefficient, 0.02; 95% CI, 0.02 to 0.02; *P* < .001; hospitals with ≤200 vs ≥500 surgical procedures per year, THR: regression coefficient, 0.11; 95% CI, 0.11 to 0.11; *P* < .001; TKR: regression coefficient, 0.08; 95% CI, 0.08 to 0.08; *P* < .001), and among patients in the highest quintile of EQ-5D-3L scores (fourth quintile vs highest quintile, THR: regression coefficient, −0.15; 95% CI, −0.15 to −0.15; *P* < .001; highest quntile vs lowest quintile, TKR: regression coefficient, −0.13; 95% CI, −0.13 to −0.13; *P* < .001) (eTable 5 and eTable 6 in the Supplement).

#### OHS and OKS Change

Greater absolute change in OHS and OKS scores at 6 months was associated with private hospitals (THR: regression coefficient, 0.75; 95% CI, 0.61 to 0.90; *P* < .001; TKR: regression coefficient, 0.73; 95% CI, 0.57 to 0.88; *P* < .001), high-volume lead surgeons (lead surgeon with ≤10 vs >150 surgical procedures per year, THR: regression coefficient, −1.03; 95% CI, −1.47 to −0.58; *P* < .001; TKR: regression coefficient, −0.54; 95% CI, −1.01 to −0.06; *P* = .03), better preoperative EQ-5D-3L scores (fourth quintile vs highest quintile, THR: regression coefficient, 3.86; 95% CI, 3.56 to 4.16; *P* < .001; highest quintile vs lowest quintile, TKR: regression coefficient, 3.77; 95% CI, 3.56 to 3.98; *P* < .001), lower CCI scores (≥3, THR: regression coefficient, −1.03; 95% CI, −1.36 to −0.71; *P* < .001; TKR: regression coefficient, −1.22; 95% CI, −1.53 to −0.91; *P* < .001), and better ASA grade (grade 4-5, THR: regression coefficient, −2.37; 95% CI, −3.41 to −1.34; *P* < .001; TKR: regression coefficient, −2.94; 95% CI, −4.19 to −1.69; *P* < .001) (eTable 7 and eTable 8 in the Supplement). Greater change in OHS was associated with bigger femoral head size (≥44 mm vs ≤28 mm: regression coefficient, 2.07; 95% CI, 0.28 to 3.86; *P* = .02) and less deprived areas (lowest quintile vs highest quintile: regression coefficient, 1.50; 95% CI, 1.30 to 1.70; *P* < .001). Patients aged 60 years or older were associated with greater change in OKS score (eg, age 70-79: regression coefficient, 2.86; 95% CI, 2.33 to 3.39; *P* < .001).

#### Complication at 6 Months

A higher probability of developing complications in the 6 months after surgery was associated with older age (≥85 years, THR: regression coefficient, 1.10; 95% CI, 0.91 to 1.28; *P* < .001; TKR: regression coefficient, 0.55; 95% CI, 0.37 to 0.73; *P* < .001), a CCI score of 3 or higher (THR: regression coefficient, 0.74; 95% CI, 0.64 to 0.83; *P* < .001; TKR: regression coefficient, 0.68; 95% CI, 0.58 to 0.77; *P* < .001), an ASA grade of 4 or 5 (THR: regression coefficient, 0.82; 95% CI, 0.59 to 1.04; *P* < .001; TKR: regression coefficient, 0.88; 95% CI, 0.62 to 1.14; *P* < .001), lower-volume hospitals (hospitals with ≤200 vs ≥500 surgical procedures per year, THR: regression coefficient, 0.12; 95% CI, 0.04 to 0.21; *P* < .001; TKR: regression coefficient, 0.09; 95% CI, 0.01 to 0.18; *P* = .03), and public hospitals (private hospitals, THR: regression coefficient, −0.08; 95% CI, −0.15 to −0.01; *P* = .03; TKR: regression coefficient, −0.10; 95% CI, −0.16 to −0.04; *P* < .001) (eTable 9 and eTable 10 in the Supplement). Hospitals conducting more surgical procedures per year correlated ecologically at the CCG level with a lower percentage of complications (THR: ρ, −0.38; TKR: ρ, −0.26), while hospitals with higher proportion of midgrade or early-career physicians correlated with higher percentage of complications (midgrade physicians, THR: ρ, 0.20; TKR: ρ, 0.19; early-career physicians, THR: ρ, 0.21; TKR: ρ, 0.19) (eTable 4 in the Supplement). For THR, thromboprophylaxis based on aspirin only was associated with complications at 6 months (regression coefficient, 0.19; 95% CI, 0.04 to 0.34; *P* = .01) (eTable 9 in the Supplement). Fewer complications were associated with minimally invasive hip replacement surgery (regression coefficient, −0.27; 95% CI, −0.43 to −0.12; *P* < .001). For TKR, private treatment centers (regression coefficient, −0.30; 95% CI, −0.42 to −0.17; *P* < .001) and unicompartmental implants (regression coefficient, 0.41; 95% CI, 0.30 to 0.52; *P* < .001) were associated with a lower percentage of complications 6 months after surgery.

### Variation in Outcomes

#### LOS and Bed-Day Costs

**Figure 2.**
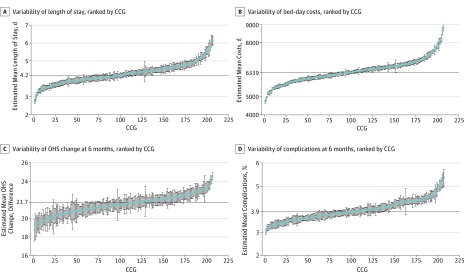
Caterpillar Plots of Patient Outcomes for Primary Hip Replacement by Health Area in England, 2014-2016 A-D, Horizontal line indicates the estimated mean value for the sample. A, Error bars indicate Poisson SEs of the mean. B-C, Error bars indicate SE of the mean. D, Error bars indicate Poisson SEs of the mean. To convert pounds sterling to US dollars, multiply by 1.23. CCG indicates clinical commissioning group; OHS, Oxford hip score.

**Figure 3.**
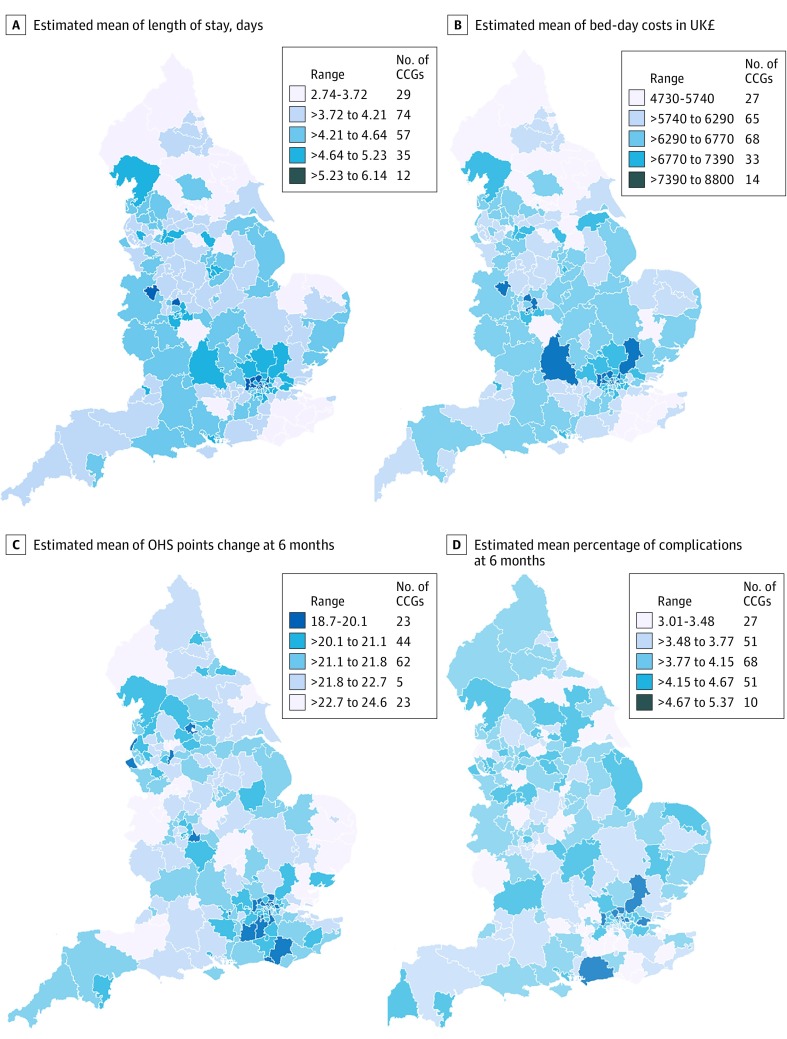
Maps of Patient Outcomes for Primary Hip Replacement Across 207 Health Areas in England, 2014-2016 To convert pounds sterling to US dollars, multiply by 1.23. CCG indicates clinical commissions group; OHS, Oxford hip score.

**Figure 4.**
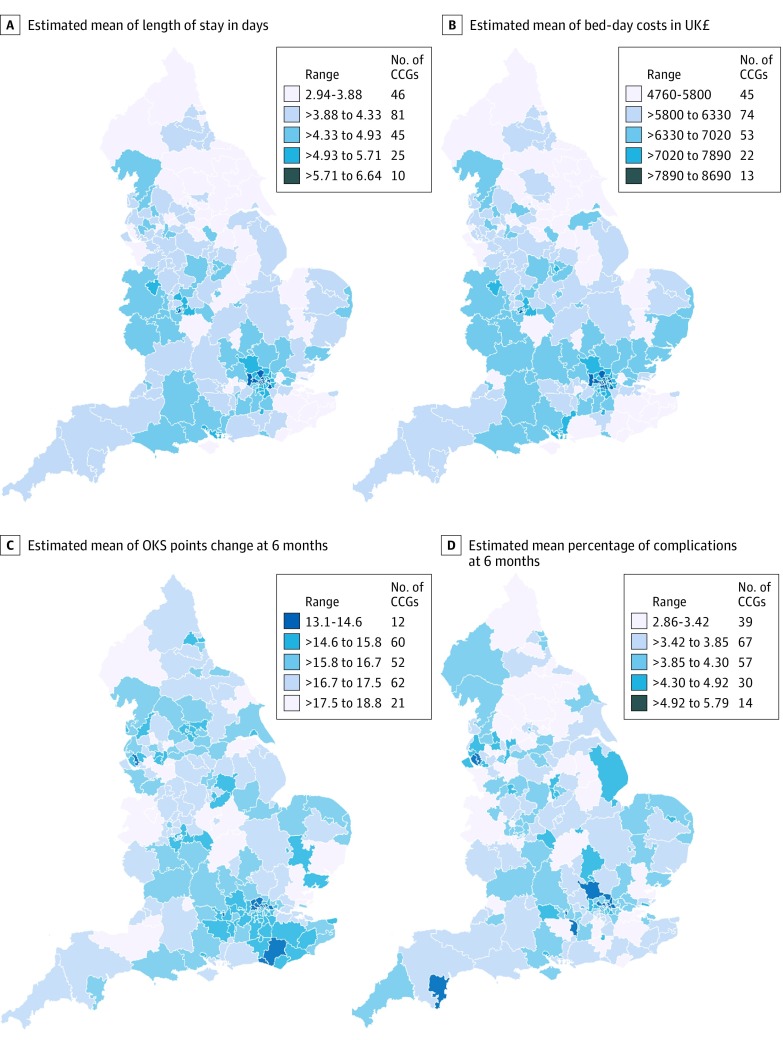
Maps of Patient Outcomes for Primary Knee Replacement Across 207 Health Areas in England, 2014-2016 To convert pounds sterling to US dollars, multiply by 1.23. CCG indicates clinical commissions group; OKS, Oxford knee score.

Observed (ie, unadjusted) mean LOS by CCG ranged from 2.5 to 6.2 days for THR and from 2.7 to 6.6 days for TKR. Fully adjusted models show that variability across CCGs remained high; for THR, 73 of 207 CCGs (35.3%) had shorter mean LOS, and 86 CCGs (41.5%) had longer mean LOS than the overall mean (Figure 2A). We also observed variability between CCGs for patients undergoing TKR, with 87 CCGs (42.0%) with shorter mean LOS and 75 CCGs (36.2%) with longer mean LOS (eFigure 2 in the Supplement). The 5 CCGs with shortest mean LOS and the 5 CCGs with the longest mean LOS appear in eTable 11 in the Supplement. Maps of England with CCG boundaries show the London region had a longer mean LOS for both THR and TKR, while North England and the East had shorter mean LOS estimates for THR and TKR (Figure 3A and Figure 4A).^14^

Mean (SD) bed-day costs for THR ranged between £4727 (£1026) ($5827 [$1265]) in NHS Scarborough and Ryedale CCG (within the Yorkshire and the Humber region) and £8800 (£1572) ($10 848 [$1938]) in NHS Hillingdon CCG (within the London region) (eTable 11 in the Supplement). Mean (SD) bed-day costs for TKR ranged between £4758 (£1096) ($5865 [$1351]) in NHS Scarborough and Ryedale CCG and £8692 (£1507) ($10 714 [$1858]) in NHS Central London CCG (eTable 11 in the Supplement). Those CCGs were consistently ranked high or low during the study period (2014-2016) for LOS and costs (eFigure 3 and eFigure 4 in the Supplement).

#### OHS and OKS Change

Observed (ie, unadjusted) mean OHS change by CCG ranged from 17.5 to 24.9 points; mean OKS change by CCG ranged from 11.2 to 19.1 points. Caterpillar plots exploring the variability of OHS change in fully adjusted models demonstrated less variability between CCGs than for OKS change, with 63 CCGs (30.4%) having lower OHS change and 45 (21.7%) having higher OHS change (Figure 2C). Variation between CCGs was greater for OKS change with 78 CCGs (37.7%) having less OKS change and 55 (26.6%) having greater OKS change (eFigure 2 in the Supplement). Mean (SD) OHS improvement ranged from 24.6 (5.3) points and 18.7 (6.2) points (Figure 3C; eTable 11 in the Supplement). Mean (SD) OKS improvement ranged from 18.8 (4.2) points to 13.1 (4.3) points (Figure 4C; eTable 11 in the Supplement). For OHS change, the same CCGs were consistently outliers during the study period, but there was variability in which CCGs were outliers for the OKS change (eFigure 3 and eFigure 4 in the Supplement).

#### Complication at 6 Months

Observed (ie, unadjusted) complications at 6 months by CCG ranged from 2.0% to 8.6% for THR and from 1.5% to 8.4% for TKR. Fully adjusted models for complications at 6 months showed 66 CCGs (31.9%) had higher complications for patients undergoing THR (Figure 2D). There was more variability for TKR, where 81 CCGs (39.1%) had higher rates of complications (eFigure 2 in the Supplement). Complications at 6 months ranged between 2.9% and 5.8% (eTable 11 in the Supplement) for patients with THR and TKR. Maps of CCGs show that the London region had a higher percentage of complications (Figure 3D and Figure 4D). Variability over the study period for complications at 6 months was consistent for the 5 CCGs with lower mean percentage of complications but changed for the 5 CCGs with a higher mean percentage of complications at 6 months (eFigure 3 and eFigure 4 in the Supplement).

## Discussion

We have previously shown^15^ that patient outcomes have been improving substantially during the past decade, with shorter mean LOS, greater reductions in pain, greater improvements in functional outcome, and fewer surgical complications. However, despite these improvements, using the most recent years of NJR data (ie, 2014-2016), we found that there is still substantial variation in patient outcomes for THR and TKR across CCG areas that remained after adjusting for patient case mix and surgical factors. Hospital organizational factors had some influence on explaining this variation, as is demonstrated in the ecological correlations at CCG level. Variation in outcomes between CCGs was greater for TKR than for THR. Length of stay had high variation between CCGs (eg, for TKR, 87 CCGs [42.0%] had shorter LOS, and 75 CCGs [36.2%] had longer). There was less variation between CCGs for OHS and OKS change outcomes (eg, 78 CCGs [37.7%] with smaller OKS change and 55 CCGs [26.6%] with larger), while there was relatively little CCG variation for complications 6 months after surgery. The substantial variation within each CCG for the OHS and OKS change outcomes was notable.

There are a large number of studies within the literature that have identified factors associated with patient outcomes for THR and TKR. A large observational study^16^ involving 10 961 primary THR and 10 260 primary TKR in the United Kingdom found that older age at surgery was associated with longer LOS (patients aged 55 years, THR: regression coefficient, 6.2; 95% CI 5.9-6.4; TKR: regression coefficient, 5.7; 95% CI, 5.5-5.9; patients aged 85 years, THR: regression coefficient, 10.6; 95% CI, 10.1-11.0; TKR: regression coefficient, 9.1; 95% CI, 8.7-9.5). Longer stays were also associated with lower socioeconomic status, and shorter stays were associated with male sex.^16^ However, LOS literature is mostly in the context of enhanced recovery interventions,^17^ where our previous work showed that older age and comorbidity were associated with longer LOS.^15^ Regarding patient case-mix variables, it has been shown that lower baseline levels of pain and functional disease severity,^3,18,19^ age,^20^ sex,^18^ obesity,^18,20^ comorbidities,^18,19^ and socioceconomic deprivation^21,22^ are all associated with patient-reported outcomes of postoperative pain and function. Less is known about factors associated with rarer outcomes, such as complications of surgery, but we have previously shown^23^ that such complications are rare and that obesity was associated with small but clinically insignificant effects. Much of this work on factors associated with the outcomes of hip and knee replacement surgery has been formally synthesized within large systematic reviews.^24^

We have previously demonstrated^25^ evidence of geographical variation and inequity in access to THR and TKR surgery for patients who underwent operations in 2002 (between 12 and 14 years before the patients in our study had their operations). However, among patients who navigate the care pathway and obtain access to joint replacement surgery, there has been little research exploring geographical variations in the outcomes of such common surgical procedures, and there is a strong need to identify modifiable process factors that are associated with variations in outcome. A previous study by Street et al^5^ used HES data to explore variation in Patient Reported Outcome Measures for THR and TKR across hospitals in England. Using multilevel regression modeling, they looked at whether patient factors (ie, age, sex, comorbidity, and socioeconomic deprivation) and hospital factors (ie, volume and teaching hospital status) were associated with health outcomes (ie, EQ-5D-3L, OHS, and OKS) and resource use (ie, LOS and hospital costs). The key findings were significant unexplained variation among hospitals in both health outcomes and resource use. This is consistent with the findings of our study; however, our research moves this forward by looking at variation in other relevant outcomes (ie, bed-day costs and complications) and at a broader range of surgical and hospital organizational factors that may be associated with geographical variation in patient outcomes, adjusted for patient case mix. Our findings suggest that such factors do not fully explain this variation. Hence, there are likely other unmeasured, historical organizational factors and processes specific to individual local hospitals that may be associated with such variation.

Birkmeyer et al^26^ suggested in a narrative review that surgical variation results mainly from differences in physician beliefs about the indications for surgery and the extent to which patient preferences are incorporated into treatment decisions, which might indicate an underuse of the procedure in some regions and/or an overuse in others.^2^ Previous research^27^ has shown that public hospitals that had a private hospital close by experienced substantial reductions in presurgery length of stay for hip and knee replacement, and the authors suggested that hospitals exposed to competition from new private entrants became more efficient. However, the negative consequence was a worsening in the complexity and case mix of patients being treated in the public hospitals, with this contributing to an increase in public hospitals’ postsurgery LOS. While policy makers may have intended this differential in healthy and less healthy (ie, straightforward and more complex) patients between public and private hospitals, there have potentially been unintended consequences. The ecological correlations at CCG level that we observed between the public and private hospitals in bed-day costs and the other outcomes could be explained by greater hospital efficiencies in the private setting but also by the changing case mix of public hospitals treating an increasing number of more complex patients, patients with poorer health, more patients with obesity, and older patients in those areas with competing private hospitals, which might explain regional variability.^27,28^ In addition, health areas with hospitals and lead surgeons performing a higher volume of joint replacement procedures per year could explain variation between regions. However, the proportion of total variance explained by the health area level was low (eg, 0.5% and 1.2% for OHS and OKS outcomes, respectively). Although we have shown that this phenomenon is unlikely to be associated with population differences, as we have accounted for patient case-mix factors, there will still be residual confounding and selection bias, particularly between patient selection at public and private hospitals, that cannot be fully accounted for by adjustment in a regression model and observational study design.

### Strengths and Limitations

Strengths of the study include use of the NJR data set, which is the largest arthroplasty data set in the world, without restricting analysis to a certain group of patients or implant providers. This allowed us to generalize the results to the English population. The NJR has near complete coverage of all arthroplasties, particularly since 2011, when the Department of Health made NJR compliance mandatory. Linkage to HES allowed us to examine a wide range of comorbidities and to link hospital organizational factors; however, analysis was restricted to England and private operations were not included in the HES data set. The large sample size allowed us to explore geographical variation in rare outcomes of rare complications.

The main limitations of the study are missing data, which were particularly prevalent for the hospital organizational factors. To overcome this, we used multiple imputation methods, but only single imputation was possible given the complexity of the multilevel regression models fitted. The main limitation of observational studies like ours is the potential for residual confounding, particularly for patient case-mix variables, owing to other measures of patient case mix not fully accounted for in our models (eg, the type of work that patients are returning to, levels of depression, availability of social support on discharge, and assumptions about weighting in the CCI) that may not reflect the relative weight of different comorbidities’ association with THR and TKR outcomes. There may also be differences in the way that surgery is performed in different units that were not captured by our data. Historically, units and regions adopt surgical practices that may influence outcome, eg, every physician in a unit uses a tourniquet or excises the fat pad in total knee replacement operations. However, this is, to our knowledge, the most thorough attempt to adjust for a very wide range of patient, surgical, and hospital factors, and given the magnitude of variation that remains, particularly for LOS, there would have to be strong residual confounding that is not correlated with the confounders already adjusted for to fully explain the remaining variability.

## Conclusions

Our models indicated that better outcomes for THR and TKR were associated with higher surgical volume by surgeon and hospital as well as private hospitals. A higher proportion of less experienced physicians by hospital was associated with poorer outcomes. The ecological correlations observed between the public and private hospitals could be explained by the changing case mix of public hospitals treating an increasing number of more complex patients.

## Notes

Supplement. **eAppendix 1.** Cost Methods **eAppendix 2.** Codes Defined in the *International Statistical Classification of Diseases and Related Health Problems, Tenth Revision* (*ICD*-*10*) Used to Identify Complications in the Hospital Episode Statistics (HES) Registry **eAppendix 3.** Operative Procedure Codes (OPCS 4.8) Used to Identify Blood-Transfusion Complication in the Hospital Episode Statistics (HES) Registry **eTable 1. **Potential Confounders of Outcome for Patients Who Underwent Planned Primary Hip or Knee Replacement From 2014 to 2016 **eTable 2. **Poisson Multilevel Models of Length of Stay for Patients Who Underwent Planned Primary Hip Replacement **eTable 3. **Poisson Multilevel Models of Length of Stay for Patients Who Underwent Planned Primary TKR/UKR **eTable 4. **Correlation Coefficients (ρ) of Hospital Organizational Factors vs Predicted Outcomes of Surgery for Patients Undergoing Primary Joint Replacement, 2014-2016, England, United Kingdom **eTable 5. **Poisson Multilevel Models of Bed-Day Costs for Patients Who Underwent Planned Primary Hip Replacement **eTable 6. **Poisson Multilevel Models of Bed-Day Costs for Patients Who Underwent Planned Primary TKR/UKR **eTable 7.** Continuous Multilevel Models of Oxford Hip Score for Patients Who Underwent Planned Primary Hip Replacement **eTable 8. **Continuous Multilevel Models of Oxford Knee Score for Patients Who Underwent Planned Primary TKR/UKR **eTable 9. **Logistic Multilevel Models of Complications at 6 Months for Patients Who Underwent Planned Primary Hip Replacement **eTable 10. **Logistic Multilevel Models of Complications at 6 Months for Patients Who Underwent Planned Primary Hip TKR/UKR **eTable 11.** Estimation of Patient Outcomes of Hip Replacement, TKR, and UKR, Adjusted for Patient, Surgical, and Hospital Organizational Factors **eFigure 1.** Flow Diagram Showing Selection of Patients for Inclusion in This Study **eFigure 2. **Caterpillar Plots of Patient Outcomes for Primary Knee Replacement by Health Area in England, 2014-2016 **eFigure 3. **Trends for 5 Worst and Best Health Areas (Clinical Commissioning Group Areas) for Primary Hip Replacement, England, 2014-2016 **eFigure 4. **Trends for 5 Worst and Best Health Areas (Clinical Commissioning Group Areas) for Primary TKR/UKR, England, 2014-2016
