# Supplementary material for: Geographical Variation in Outcomes of Primary Hip and Knee Replacement
Source: JAMA Netw Open. 2019 Oct 30;2(10):e1914325. doi: 10.1001/jamanetworkopen.2019.14325 (PMC6824227; doi:10.1001/jamanetworkopen.2019.14325)
Supplement: Supplement. — eAppendix 1. Cost Methods eAppendix 2. Codes Defined in the International Statistical Classification of Diseases and Related Health Problems, Tenth Revision (ICD-10) Used to Identify Complications in the Hospital Episode Statistics (HES) Registry eAppendix 3. Operative Procedure Codes (OPCS 4.8) Used to Identify Blood-Transfusion Complication in the Hospital Episode Statistics (HES) Registry eTable 1. Potential Confounders of Outcome for Patients Who Underwent Planned Primary Hip or Knee Replacement From 2014 to 2016 eTable 2. Poisson Multilevel Models of Length of Stay for Patients Who Underwent Planned Primary Hip Replacement eTable 3. Poisson Multilevel Models of Length of Stay for Patients Who Underwent Planned Primary TKR/UKR eTable 4. Correlation Coefficients (ρ) of Hospital Organizational Factors vs Predicted Outcomes of Surgery for Patients Undergoing Primary Joint Replacement, 2014-2016, England, United Kingdom eTable 5. Poisson Multilevel Models of Bed-Day Costs for Patients Who Underwent Planned Primary Hip Replacement eTable 6. Poisson Multilevel Models of Bed-Day Costs for Patients Who Underwent Planned Primary TKR/UKR eTable 7. Continuous Multilevel Models of Oxford Hip Score for Patients Who Underwent Planned Primary Hip Replacement eTable 8. Continuous Multilevel Models of Oxford Knee Score for Patients Who Underwent Planned Primary TKR/UKR eTable 9. Logistic Multilevel Models of Complications at 6 Months for Patients Who Underwent Planned Primary Hip Replacement eTable 10. Logistic Multilevel Models of Complications at 6 Months for Patients Who Underwent Planned Primary Hip TKR/UKR eTable 11. Estimation of Patient Outcomes of Hip Replacement, TKR, and UKR, Adjusted for Patient, Surgical, and Hospital Organizational Factors eFigure 1. Flow Diagram Showing Selection of Patients for Inclusion in This Study eFigure 2. Caterpillar Plots of Patient Outcomes for Primary Knee Replacement by Health Area in England, 2014-2016 eFigure 3. Trends for 5 Worst and [file jamanetwopen-2-e1914325-s001.pdf]

Supplementary Online Content

Garriga C, Leal J, Sánchez-Santos MT, et al. Geographical variation in outcomes of primary hip and knee replacement. *JAMA Netw Open*. 2019;2(10):e1914325. doi:10.1001/jamanetworkopen.2019.14325

eAppendix 1. Cost Methods

eAppendix 2. Codes Defined in the *International Statistical Classification of Diseases and Related Health Problems, Tenth Revision* (*ICD-10*) Used to Identify Complications in the Hospital Episode Statistics (HES) Registry

eAppendix 3. Operative Procedure Codes (OPCS 4.8) Used to Identify Blood-Transfusion Complication in the Hospital Episode Statistics (HES) Registry

eTable 1. Potential Confounders of Outcome for Patients Who Underwent Planned Primary Hip or Knee Replacement From 2014 to 2016

eTable 2. Poisson Multilevel Models of Length of Stay for Patients Who Underwent Planned Primary Hip Replacement

eTable 3. Poisson Multilevel Models of Length of Stay for Patients Who Underwent Planned Primary TKR/UKR

eTable 4. Correlation Coefficients (ρ) of Hospital Organizational Factors vs Predicted Outcomes of Surgery for Patients Undergoing Primary Joint Replacement, 2014-2016, England, United Kingdom

eTable 5. Poisson Multilevel Models of Bed-Day Costs for Patients Who Underwent Planned Primary Hip Replacement

eTable 6. Poisson Multilevel Models of Bed-Day Costs for Patients Who Underwent Planned Primary TKR/UKR

eTable 7. Continuous Multilevel Models of Oxford Hip Score for Patients Who Underwent Planned Primary Hip Replacement

eTable 8. Continuous Multilevel Models of Oxford Knee Score for Patients Who Underwent Planned Primary TKR/UKR

eTable 9. Logistic Multilevel Models of Complications at 6 Months for Patients Who Underwent Planned Primary Hip Replacement

eTable 10. Logistic Multilevel Models of Complications at 6 Months for Patients Who Underwent Planned Primary Hip TKR/UKR

eTable 11. Estimation of Patient Outcomes of Hip Replacement, TKR, and UKR, Adjusted for Patient, Surgical, and Hospital Organizational Factors

© 2019 Garriga C et al. *JAMA Network Open.*

eFigure 1. Flow Diagram Showing Selection of Patients for Inclusion in This Study

eFigure 2. Caterpillar Plots of Patient Outcomes for Primary Knee Replacement by Health Area in England, 2014-2016

eFigure 3. Trends for 5 Worst and Best Health Areas (Clinical Commissioning Group Areas) for Primary Hip Replacement, England, 2014-2016

eFigure 4. Trends for 5 Worst and Best Health Areas (Clinical Commissioning Group Areas) for Primary TKR/UKR, England, 2014-2016

This supplementary material has been provided by the authors to give readers additional information about their work.

© 2019 Garriga C et al. *JAMA Network Open.*

eAppendix 1. Cost Methods

Objective

We aimed to estimate the trend in National Health Service (NHS) expenditure over time, reflecting the change in length of stay (LOS) observed.

Grouper and reference cost methods

Using Hospital Episode Statistics (HES) data for the same group of patients as for LOS, we generated healthcare resource use group (HRG) classifications for the index episode for each patient using the 2015/16 NHS reference costs grouper [1], which were subsequently used to estimate inpatient costs per patient using NHS reference costs from 2015/16 [2]. In order to estimate the mean change in NHS expenditure we estimated an adjusted average bed day cost.

Estimating the adjusted average bed day cost

For each HRG we estimated the average cost per bed day (defined as any part of a day spent in hospital) by dividing the total cost of the index episodes for that HRG by the total number of bed days for that HRG. This generated a single average bed day cost per HRG.

For each patient we estimated the adjusted episode cost by multiplying their LOS (bed days) by the average bed day cost for the HRG that they had been assigned by the NHS reference costs grouper [1]. Therefore, instead of assigning the same unit cost to all patients with the same HRG who had a LOS below the trim point, the adjusted cost differed according to a patient's LOS, even if that LOS was below the trim point for the HRG. Using this method we were able to estimate the average difference in true NHS expenditure even when the LOS was below the trim point.

The 2015/16 grouper and reference costs [1,2] were used to estimate costs for all patients in all years, as there are differences in the methodologies used for HRG classification in

© 2019 Garriga C et al. *JAMA Network Open.*

different cost years [3]. This prevents a like-for-like comparison between years if different groupers and/or costs are used.

Costs were estimated for a total of 173,106 patients undergoing hip replacement and 210,723 total and unicompartmental knee replacements.

References for Appendix 1

1. HRG4+ 2015/16 Reference Costs Grouper. Copyright © 2015 Health and Social Care Information Centre. Grouper version: RC 15/16. Implementation version: 1516.RC.8
2. Department of Health. NHS reference costs 2015 to 2016. https://www.gov.uk/government/publications/nhs-reference-costs-2015-to-2016
3. Reference Costs 2015-16. Department of Health. https://assets.publishing.service.gov.uk/government/uploads/system/uploads/attachment_data/file/577083/Reference_Costs_2015-16.pdf.

© 2019 Garriga C et al. *JAMA Network Open.*

eAppendix 2. Codes Defined in the International Statistical Classification of Diseases and Related Health Problems, Tenth Revision (ICD-10) Used to Identify Complications in the Hospital Episode Statistics (HES) Registry

Stroke: I60.X, "Subarachnoid haemorrhage"; I61.0, "Intracerebral haemorrhage in hemisphere, subcortical"; I61.1, "Intracerebral haemorrhage in hemisphere, cortical"; I61.2, "Intracerebral haemorrhage in hemisphere, unspecified"; I61.3, "Intracerebral haemorrhage in brain stem"; I61.4, "Intracerebral haemorrhage in cerebellum"; I61.5, "Intracerebral haemorrhage, intraventricular"; I61.6, "Intracerebral haemorrhage, multiple localized"; I61.8, "Other intracerebral haemorrhage"; I61.9, "Intracerebral haemorrhage, unspecified"; I63.0, "Cerebral infarction due to thrombosis of precerebral arteries"; I63.1, "Cerebral infarction due to embolism of precerebral arteries"; I63.2, "Cerebral infarction due to unspecified occlusion or stenosis of precerebral arteries"; I63.3, "Cerebral infarction due to thrombosis of cerebral arteries"; I63.4, "Cerebral infarction due to embolism of cerebral arteries"; I63.5, "Cerebral infarction due to unspecified occlusion or stenosis of cerebral arteries"; I63.6, "Cerebral infarction due to cerebral venous thrombosis, nonpyogenic"; I63.8, "Other cerebral infarction"; I63.9, "Cerebral infarction, unspecified"; and I64.X, "Stroke, not specified as haemorrhage or infarction".

Respiratory infection: J12.X, "Viral pneumonia, not elsewhere classified: bronchopneumonia due to viruses other than influenza viruses"; J13, "Pneumonia due to *Streptococcus pneumoniae*"; J14, "Pneumonia due to *Haemophilus influenzae*"; J15.X, "Bacterial pneumonia, not elsewhere classified: bronchopneumonia due to bacteria other than *S. pneumoniae* and *H. influenzae*"; J18.0, "Bronchopneumonia, unspecified. Excluding bronchiolitis"; J18.1, "Lobar pneumonia, unspecified"; J18.2, "Hypostatic pneumonia, unspecified"; J18.8, "Other pneumonia, organism unspecified"; J18.9, "Pneumonia, unspecified"; J22, "Unspecified acute lower respiratory infection"; J44.0, "Chronic

© 2019 Garriga C et al. *JAMA Network Open.*

obstructive pulmonary disease with acute lower respiratory infection. Excluding with influenza"; J44.1, "Chronic obstructive pulmonary disease with acute exacerbation, unspecified"; J69.0, "Pneumonitis due to food and vomit. Excluding Mendelson syndrome"; J69.1, "Pneumonitis due to oils and essences"; J69.8, "Pneumonitis due to other solids and liquids. Pneumonitis due to aspiration of blood"; and J85.1, "Abscess of lung with pneumonia. Excluding with pneumonia due to specified organism".

Acute myocardial infarction: I21.0, "Acute transmural myocardial infarction of anterior wall"; I21.1, "Acute transmural myocardial infarction of inferior wall"; I21.2, "Acute transmural myocardial infarction of other sites"; I21.3, "Acute transmural myocardial infarction of unspecified site"; I21.4, "Acute subendocardial myocardial infarction"; and I21.9, "Acute myocardial infarction, unspecified".

Pulmonary embolism/deep vein thrombosis: I80.1, "Phlebitis and thrombophlebitis of superficial vessels of lower extremities"; I80.1, "Phlebitis and thrombophlebitis of femoral vein"; I80.3, "Phlebitis and thrombophlebitis of other deep vessels of lower extremities"; I26.0, "Pulmonary embolism with mention of acute cor pulmonale"; and I26.9, "Pulmonary embolism without mention of acute cor pulmonale".

Urinary tract infection: N30.0, "Acute cystitis. Excluding irradiation cystitis and trigonitis"; and N39.0, "Urinary tract infection, site not specified".

Wound disruption: T81.3, "Disruption of operation wound, not elsewhere classified".

Surgical site infection: T81.4, "Infection following a procedure, not elsewhere classified".

Fracture after implant: M96.6, "Fracture of bone following insertion of orthopaedic implant, joint prosthesis, or bone plate. Excluding complication of internal orthopaedic devices, implants or grafts".

© 2019 Garriga C et al. *JAMA Network Open.*

Complication of prosthesis: T84.0, “Mechanical complication of internal joint prosthesis”.

Neurovascular injury: T81.2, “Accidental puncture and laceration during a procedure, not elsewhere classified. Accidental perforation of: blood vessel, nerve or organ by: catheter, endoscope, instrument or probe during a procedure”.

Acute renal failure: N17.0, “Acute renal failure with tubular necrosis”; N17.1, “Acute renal failure with acute cortical necrosis”; N17.2, “Acute renal failure with medullary necrosis”; N17.8, “Other acute renal failure”; and N17.9, “Acute renal failure, unspecified”.

© 2019 Garriga C et al. *JAMA Network Open.*

eAppendix 3. Operative Procedure Codes (OPCS 4.8) Used to Identify Blood-Transfusion Complication in the Hospital Episode Statistics (HES) Registry

X33.2, “Intravenous blood transfusion of packed cells”; X33.3, “Intravenous blood transfusion of platelets”; X33.8, “Other specified other blood transfusion”; X33.9, “Unspecified other blood transfusion”; X33.1, “Intra-arterial blood transfusion”; X33.7, “Autologous transfusion of red blood cells”; X34.1, “Transfusion of coagulation factor”; X34.2, “Transfusion of plasma not elsewhere classified”; X34.3, “Transfusion of serum not elsewhere classified”; and X34.4, “Transfusion of blood expander”.

© 2019 Garriga C et al. *JAMA Network Open.*

eTable 1. Potential Confounders of Outcome for Patients Who Underwent Planned Primary Hip or Knee Replacement From 2014 to 2016

**Factors**	**Additional information**	**Source**
Patient factors		
Calendar year	2014-2016	NJR
Age (years)	<50, 50-59, 60-69, 70-79, 80-84, ≥85	NJR
Sex	Man; Woman	NJR
Body mass index (kg/m^2)	16-60	NJR
Pre-operative ASA physical function score	Grade 1, fit and healthy; grade 2, mild disease; grade 3, incapacitating disease; grades 4 and 5, life-threatening disease or expected to die within 24 hours	NJR
IMD (quintile groupings)	Grouped from "IMD Decile Group" variable using the IMD Overall Ranking to identify which one of five groups a Super Output Area belongs to (rank of 32482 is the least deprived, and 1 the most deprived). Least deprived 20%, 25987-32482; Less deprived 20-40%, 19490-25986; Less deprived 40-60%, 12994-19489; More deprived 20-40%, 6497-12993; Most deprived 20%, 1-6496	HES
Rural/Urban Indicator	Context from the original variable (sparse or less sparse) is ignored and variable is grouped into urban >=10,000 habitants; town and fringe; village/isolated	HES
Primary indication	Osteoarthritis; and osteoarthritis plus other indication.<br>Other indication for hip replacement: Avascular Necrosis, Other Inflammatory Arthropathy, Acute Trauma Neck of Femur, Chronic Trauma, Previous	NJR

© 2019 Garriga C et al. *JAMA Network Open.*

	Surgery non-trauma Related, Ankylosing Spondylitis, Congenital Dislocation Dysplasia of Hip, Failed Hemi-Arthroplasty, Failed Internal Fixation, Fractured Acetabulum, Fractured Neck of Femur, Other Hip Trauma, Perthes, Previous Arthrodesis, Psoriatic Arthropathy, Seropositive Rheumatoid Arthritis, Seronegative Rheumatoid Arthritis, Slipped Upper Femoral Epiphysis (SUFE), Previous Infection, Trauma, Previous Hip Trauma not specified, Metastatic Cancer or Malignancy, Skeletal Dysplasia, or Other.<br>Other indication for knee replacement: Avascular Necrosis, Other Inflammatory Arthropathy, Previous Trauma, Failed Internal Fixation, Previous Arthrodesis, Seropositive Rheumatoid Arthritis, Seronegative Rheumatoid Arthritis, Rheumatoid Arthritis, Previous Infection, Trauma, Previous knee Trauma not specified, or Other.	
Charlson score	Grouped into 4 groups (none=0, mild=1, moderate=2, severe≥3)	HES
Baseline OHS/baseline OKS	0 the worst possible and 48 the best possible score	PROMs
EQ-5D-3L (quintile groupings)	-0.594 the worst possible and 1 the best possible score grouped in quintiles (hips: -0.594 to -0.016; -0.017 to 0.100; 0.101 to 0.586; 0.587 to 0.690; 0.691 to 1; knees: -0.594 to 0.054; 0.055 to 0.158; 0.159 to 0.610; 0.620 to 0.690; 0.691 to 1)	PROMs
Surgical factors		
Lead surgeon experience	Consultant with ≥8 training years, and surgeons with <8 (including Associate Specialist [4 training years], Foundation doctor (FY1 and FY2) [2 training years],	NJR

© 2019 Garriga C et al. *JAMA Network Open.*

	Specialty trainees years 1 and 2 (ST1 and ST2) [4 training years], Fellow, House Officer, Senior House Officer [minimum 2 years; often more], Specialist Registrar (SPR) [minimum 6 years], specialty general surgical training (ST3-8) [minimum 6 years], Specialty Doctor/Specialty and associate specialist (SAS) doctors [non-training roles where the doctor has at least four years of postgraduate training], Staff grade/ Clinical Assistant, Other)	
Surgical volume per lead surgeon (number of surgeries per year)	Grouped into 6 categories (≤10; 11-50; 51-75; 76-100; 101-150; >150)	NJR
Surgical volume per unit (number of surgeries per year)	Grouped into 5 categories (<200; 200-299; 300-399; 400-499; ≥500)	NJR
Minimally invasive surgery	Yes; No	NJR
Thromboprophylaxis	Grouped into 4 categories: None; Aspirin only; LMWH (with/without pentasaccharide, warfarin, direct thrombin inhibitor, direct factor Xa inhibitor, other); Other except LMWH)	NJR
Mechanical prophylaxis	Grouped into 2 categories: None; Any (venous foot pump, elastic compression stockings, intermittent calf compression)	NJR
Anaesthetic type	General, regional - epidural, regional - nerve block, and regional - spinal (intrathecal)	NJR
Type of approach	<u>In hip replacement</u>: posterior, other [anterior, antero-lateral, hardinge, lateral, trochanteric osteotomy, other]<br><u>In knee replacement</u>: lateral parapatellar, medial parapatellar, mid-vastus, sub-vastus, other	NJR
Hip bone graft		

Femoral bone graft	None; Any	NJR
Cup bone graft	None; Any	NJR
Knee bone graft		
Femoral bone graft	None; Any	NJR
Tibial bone graft	None; Any	NJR
Hip implant fixation		
Cup fixation	Cementless; Cemented	NJR
Stem fixation except THR	Cementless hip replacement stem; Cemented hip replacement stem; THR or missing	NJR
Stem fixation except resurfacing	Cementless THR stem; Cemented THR stem; resurfacing or missing	NJR
Knee implant fixation		
Femoral fixation	Cementless; Cemented	NJR
Tibial fixation	Cementless; Cemented	NJR
Type of hip implant		
Bearing surface	Grouped into 5 categories: MoM; MoP; CoC; CoP; and CoM or MoC or missing	NJR
Femoral head size (mm)	Grouped into 4 categories: ≤28, 32, 36-42, and ≥44	NJR
Type of knee implant	Grouped into 2 categories: Total knee replacement; and Unicompartmental knee replacement.	NJR
Hospital organisation factors		
Unit type	Public hospital; Private hospital; and Independent sector (private) - treatment centre	NJR
FTE of speciality groups on trauma and orthopaedic surgery	0-24; 25-29; 30-39; 40-49; >50	HCHS
consultants FTE	0-24; 25-29; 30-39; 40-49; >50	HCHS
middle grade doctor FTE	0-24; 25-29; 30-39; 40-49; >50	HCHS
early career doctor FTE	0-24; 25-29; 30-39; 40-49; >50	HCHS

© 2019 Garriga C et al. *JAMA Network Open.*

Total beds available overnight	0-349; 350-499; 500-699; 700-999; ≥1000	KH03 – NHS England
Total beds available overnight for trauma and orthopaedic surgery	0-34; 35-49; 50-69; 70-99; ≥100	KH03 – NHS England
Total beds available overnight for rehabilitation	0; >0-10; 11-20; ≥20	KH03 – NHS England
Operating theatres	<10; 10-14; 15-19; 20-24; ≥25	NHS England
Dedicated day case operating theatres	0; 1-2; 3-4; 5-6; ≥7	NHS England

UK National Joint Registry, NJR; American Society of Anesthesiologists, ASA; Index of multiple deprivation, IMD; hospital episode statistics, HES; Oxford hip score, OHS; Oxford knee score, OKS; Patient Reported Outcome Measures, PROMs; European Quality of life-five domain, EQ-5D-3L; low-molecular-weight heparin, LMWH; total hip replacement, THR; metal-on-metal, MoM; metal-on-polyethylene, MoP; ceramic-on-ceramic, CoC; ceramic-on-polyethylene, CoP; ceramic-on-metal, CoM; metal-on-ceramic, MoC; full time equivalent, FTE (proportion of full time contracted hours); NHS Hospital & Community Health Service, HCHS (monthly workforce statistics); quarterly collection from all NHS organisations that operate beds, KH03 – NHS England.

© 2019 Garriga C et al. *JAMA Network Open.*

eTable 2. Poisson Multilevel Models of Length of Stay for Patients Who Underwent Planned Primary Hip Repla[illegible]

		Patient model			Surgical model			Hospital[illegible]	
	Categories	Coef.	95% CI	P	Coef.	95% CI	P	Coef.	
Intercept		1.16	[1.13-1.20]	<0.01	1.17	[1.14-1.21]	<0.01	1.13	
Patient factors									
Calendar year (2016)	2015	0.09	[0.08-0.09]	<0.01	0.08	[0.07-0.08]	<0.01	0.07	
	2016	0.05	[0.04-0.05]	<0.01	0.04	[0.03-0.04]	<0.01	0.03	
Age (<50 years)	50-59	0.01	[<0.01-0.03]	0.12	0.01	[-0.01-0.02]	0.29	0.02	
	60-69	0.06	[0.05-0.07]	<0.01	0.05	[0.03-0.06]	<0.01	0.07	
	70-79	0.20	[0.19-0.22]	<0.01	0.17	[0.16-0.19]	<0.01	0.19	
	80-84	0.42	[0.41-0.44]	<0.01	0.38	[0.37-0.40]	<0.01	0.39	
	≥85	0.63	[0.61-0.64]	<0.01	0.59	[0.57-0.60]	<0.01	0.59	
Sex (woman)	man	-0.09	[-0.09-(-0.08)]	<0.01	-0.09	[-0.09-(-0.08)]	<0.01	-0.09	[illegible]
BMI, (kg/m^2)		<0.01	[<0.01-<0.01]	<0.01	<0.01	[<0.01-<0.01]	<0.01	<0.01	[illegible]
Pre-operative ASA physical function score (Mild disease not incapacitating, grade 2)	Fit and healthy (grade 1)	-0.06	[-0.07-(-0.05)]	<0.01	-0.05	[-0.06-(-0.05)]	<0.01	-0.05	[illegible]

© 2019 Garriga C et al. *JAMA Network Open.*

	Incapacitating systemic disease (grade 3)	0.22	[0.21-0.22]	<0.01	0.20	[0.20-0.21]	<0.01	0.17	[0.17-0.18]	<0.01
	Life threatening disease or Expected to die within 24h (grades 4 and 5)	0.43	[0.40-0.46]	<0.01	0.41	[0.38-0.44]	<0.01	0.36	[0.33-0.39]	<0.01
IMD (Most deprived 20%)	Least deprived 20%	-0.04	[-0.05-(-0.03)]	<0.01	-0.03	[-0.05-(-0.02)]	<0.01	-0.02	[-0.04-(-0.01)]	<0.01
	Less deprived 20-40%	-0.03	[-0.04-(-0.02)]	<0.01	-0.03	[-0.04-(-0.01)]	<0.01	-0.02	[-0.03-(-0.01)]	<0.01
	Less deprived 40-60%	-0.02	[-0.03-(-0.01)]	<0.01	-0.02	[-0.03-(-0.01)]	<0.01	-0.02	[-0.03-(-0.01)]	<0.01
	More deprived 20-40%	-0.01	[-0.02-<0.01]	0.07	-0.01	[-0.02-<0.01]	0.06	-0.01	[-0.02-<0.01]	0.11
Rural/Urban indicator (Urban >=10,000 habitants)	Town and fringe	-0.01	[-0.02-<0.01]	0.08	-0.01	[-0.02-<0.01]	0.14	<0.01	[-0.02-0.01]	0.44

© 2019 Garriga C et al. *JAMA Network Open.*

	Village/isolated	-0.02	[-0.03-(-0.01)]	<0.01	-0.02	[-0.03-(-0.01)]	<0.01	-0.01	[-0.02-<0.01]	0.04
Primary indication (Osteoarthritis)	Osteoarthritis and Other*	0.18	[0.17-0.20]	<0.01	0.17	[0.16-0.19]	<0.01	0.14	[0.13-0.15]	<0.01
Charlson score (None, 0)	Mild (1)	0.08	[0.07-0.08]	<0.01	0.08	[0.07-0.08]	<0.01	0.07	[0.06-0.07]	<0.01
	Moderate (2)	0.19	[0.18-0.19]	<0.01	0.18	[0.17-0.19]	<0.01	0.16	[0.15-0.17]	<0.01
	Severe (≥3)	0.34	[0.33-0.35]	<0.01	0.33	[0.32-0.34]	<0.01	0.30	[0.29-0.32]	<0.01
EQ-5D-3L (Highest quintile)	Lowest quintile	-0.13	[-0.14-(-0.12)]	<0.01	-0.13	[-0.14-(-0.12)]	<0.01	-0.12	[-0.13-(-0.12)]	<0.01
	2nd quintile	-0.17	[-0.18-(-0.16)]	<0.01	-0.16	[-0.17-(-0.16)]	<0.01	-0.16	[-0.17-(-0.15)]	<0.01
	3rd quintile	-0.20	[-0.21-(-0.19)]	<0.01	-0.19	[-0.20-(-0.18)]	<0.01	-0.18	[-0.19-(-0.17)]	<0.01
	4th quintile	-0.24	[-0.25-(-0.23)]	<0.01	-0.23	[-0.24-(-0.22)]	<0.01	-0.22	[-0.23-(-0.21)]	<0.01
Surgical factors										
Lead surgeon experience (Consultant)	Other**				0.09	[0.08-0.10]	<0.01	0.02	[0.01-0.03]	<0.01
Surgical volume per lead surgeon (>150 surgeries per year)	≤10				0.11	[0.09-0.13]	<0.01	0.04	[0.03-0.06]	<0.01

© 2019 Garriga C et al. *JAMA Network Open.*

	11-50				0.08	[0.07-0.09]	<0.01	0.02	[0.02-0.03]	<0.01
	51-75				0.05	[0.04-0.06]	<0.01	0.01	[<0.01-0.02]	0.01
	76-100				0.03	[0.02-0.04]	<0.01	<0.01	[-0.01-0.01]	0.96
	101-150				0.03	[0.02-0.04]	<0.01	0.01	[<0.01-0.02]	0.08
Surgical volume per unit (Surgeries per year, ≥500)	≤200				0.05	[0.04-0.06]	<0.01	0.14	[0.13-0.15]	<0.01
	200-299				<0.01	[-0.01-0.01]	0.56	0.06	[0.05-0.07]	<0.01
	300-399				-0.04	[-0.06-(-0.03)]	<0.01	0.01	[<0.01-0.03]	0.02
	400-499				-0.04	[-0.06-(-0.03)]	<0.01	0.05	[0.04-0.07]	<0.01
Minimally invasive surgery (No)	Yes				-0.05	[-0.07-(-0.04)]	<0.01	-0.03	[-0.05-(-0.02)]	<0.01
Thromboprophylaxis (LMWH +/-Other)	None				<0.01	[-0.02-0.03]	0.91	0.03	[<0.01-0.05]	0.04
	Aspirin only				-0.03	[-0.05-(-0.02)]	<0.01	-0.02	[-0.04-(-0.01)]	0.01
	Other (no LMWH)				-0.02	[-0.03-(-0.02)]	<0.01	<0.01	[<0.01-0.01]	0.42
Mechanical prophylaxis (any)	None				0.08	[0.06-0.09]	<0.01	0.03	[0.01-0.04]	<0.01

© 2019 Garriga C et al. *JAMA Network Open.*

General anaesthesia (No)	Yes				0.04	[0.03-0.05]	<0.01	0.04	[0.03-0.04]	<0.01
Regional – epidural anaesthesia (No)	Yes									
Regional – nerve block anaesthesia (No)	Yes				0.05	[0.04-0.06]	<0.01	0.04	[0.03-0.05]	<0.01
Regional – spinal anaesthesia (No)	Yes				-0.03	[-0.04-(-0.03)]	<0.01	-0.03	[-0.04-(-0.02)]	<0.01
Type of approach (Posterior)	Anterior, antero-lateral, hardinge, lateral, trochanteric osteotomy, other				0.06	[0.05-0.07]	<0.01	0.06	[0.06-0.07]	<0.01
Femoral bone graft (No)	Yes				0.06	[0.03-0.10]	<0.01	0.06	[0.03-0.10]	<0.01
Cup bone graft (No)	Yes				0.07	[0.06-0.08]	0.68	0.05	[0.04-0.07]	<0.01

© 2019 Garriga C et al. *JAMA Network Open.*

Primary cup fixation (Cementless)	Cemented				-0.01	[-0.02-(-0.01)]	<0.01	-0.01	[-0.02-<0.01]	<0.01
Type of primary stem fixation (Cemented THR stem)	Not available or Resurfacing				0.01	[-0.03-0.04]	0.89	0.03	[-0.01-0.06]	0.13
	Cementless THR stem				-0.07	[-0.08-(-0.07)]	1.00	-0.04	[-0.05-(-0.04)]	<0.01
Bearing surface (MoP)	MoM				-0.01	[-0.10-0.09]	0.89	-0.07	[-0.17-0.02]	0.14
	CoC				<0.01	[-0.01-0.01]	1.00	-0.03	[-0.04-(-0.02)]	<0.01
	CoP				-0.02	[-0.03-(-0.02)]	<0.01	-0.04	[-0.04-(-0.03)]	<0.01
	CoM or MoC or unknown				0.10	[0.08-0.13]	<0.01	0.08	[0.06-0.11]	<0.01
Femoral head size (≤28mm)	32				<0.01	[-0.01-0.01]	0.51	-0.01	[-0.01-<0.01]	0.10
	36-42				0.03	[0.02-0.04]	<0.01	0.02	[0.01-0.03]	<0.01
	≥44				-0.03	[-0.11-0.06]	<0.01	-0.04	[-0.12-0.05]	0.37
Hospital organisation factors										

© 2019 Garriga C et al. *JAMA Network Open.*

Unit type (Public hospital)	Private hospital							-0.22	[-0.23-(-0.21)]	<0.01
	Independent sector (private) – treatment centre							-0.40	[-0.41-(-0.38)]	<0.01
FTE of speciality groups on trauma and orthopaedic surgery (0-24 FTEs)	25-29							0.01	[-0.01-0.02]	0.40
	30-39							0.03	[0.02-0.04]	<0.01
	40-49							0.03	[0.01-0.04]	<0.01
	>50							0.04	[0.03-0.05]	<0.01
Consultant FTE (0-24 FTEs)	25-29							0.03	[0.02-0.04]	<0.01
	30-39							0.04	[0.03-0.05]	<0.01
	40-49							0.03	[0.01-0.04]	<0.01
	>50							0.04	[0.02-0.06]	<0.01

© 2019 Garriga C et al. *JAMA Network Open.*

middle grade doctor FTE (0-24 FTEs)	25-29							0.01	[<0.01-0.02]	0.15
	30-39							<0.01	[-0.01-0.01]	0.77
	40-49							0.01	[<0.01-0.02]	0.10
	>50							0.04	[0.03-0.05]	<0.01
early career doctor FTE (0-24 FTEs)	25-29							-0.01	[-0.03-0.01]	0.39
	30-39							-0.05	[-0.09-(-0.01)]	0.01
Total beds available overnight (0-349)	350-499							-0.04	[-0.05-(-0.02)]	<0.01
	500-699							-0.03	[-0.04-(-0.01)]	<0.01
	700-999							-0.05	[-0.06-(-0.03)]	<0.01
	≥1000							-0.09	[-0.11-(-0.07)]	<0.01
Total beds available overnight for trauma and orthopaedic surgery (0-34)	35-49							0.02	[0.01-0.03]	<0.01
	50-69							0.06	[0.05-0.07]	<0.01
	70-99							0.11	[0.09-0.12]	<0.01

© 2019 Garriga C et al. *JAMA Network Open.*

	≥100							0.16	[0.14-0.17]	<0.01
Total beds available overnight for rehabilitation (0)	>0-10							0.01	[-0.01-0.02]	0.38
	11-20							0.01	[<0.01-0.03]	0.02
	≥20							0.07	[0.06-0.08]	<0.01
Operating theatres (<10)	10-14							0.01	[-0.01-0.02]	0.27
	15-19							-0.02	[-0.04-<0.01]	0.01
	20-24							-0.09	[-0.11-(-0.07)]	<0.01
	≥25							-0.06	[-0.08-(-0.04)]	<0.01
Dedicated day case operating theatres (≥7)	0							0.09	[0.08-0.10]	<0.01
	1-2							0.06	[0.05-0.07]	<0.01
	3-4							0.07	[0.06-0.08]	<0.01
	5-6							0.04	[0.03-0.05]	<0.01

American Society of Anesthesiologists, ASA; Index of multiple deprivation, IMD; European Quality of life-five domain, EQ-5D-3L; low-molecular-weight heparin, LMWH; total hip replacement, THR; metal-on-metal, MoM; metal-on-polyethylene, MoP; ceramic-on-ceramic, CoC; ceramic-on-polyethylene, CoP; ceramic-on-metal, CoM; metal-on-ceramic, MoC; full time equivalent, FTE (proportion of full time contracted hours).

© 2019 Garriga C et al. *JAMA Network Open.*

eTable 3. Poisson Multilevel Models of Length of Stay for Patients Who Underwent Planned Primary TKR/UKR

		Patient model			Surgical model			Hospital organisation model		
	Categories	Coef.	95% CI	P	Coef.	95% CI	P	Coef.	95% CI	P
Intercept		1.08	[1.05-1.12]	<0.01	0.83	[0.79-0.87]	<0.01	1.00	[0.96-1.03]	<0.01
Patient factors										
Calendar year (2016)	2015	0.08	[0.08-0.09]	<0.01	0.08	[0.07-0.08]	<0.01	0.07	[0.06-0.08]	<0.01
	2016	0.04	[0.04-0.05]	<0.01	0.04	[0.03-0.04]	<0.01	0.03	[0.03-0.04]	<0.01
Age (<50 years)	50-59	-0.01	[-0.03-<0.01]	0.11	-0.04	[-0.06-(-0.02)]	<0.01	-0.03	[-0.05-(-0.01)]	<0.01
	60-69	0.04	[0.02-0.06]	<0.01	-0.01	[-0.03-0.01]	0.33	0.01	[-0.01-0.03]	0.36
	70-79	0.17	[0.15-0.19]	<0.01	0.11	[0.09-0.13]	<0.01	0.12	[0.11-0.14]	<0.01
	80-84	0.38	[0.36-0.40]	<0.01	0.31	[0.29-0.33]	<0.01	0.32	[0.30-0.34]	<0.01
	≥85	0.56	[0.54-0.58]	<0.01	0.49	[0.48-0.51]	<0.01	0.49	[0.47-0.51]	<0.01
Sex (woman)	man	-0.05	[-0.05-(-0.04)]	<0.01	-0.04	[-0.05-(-0.04)]	<0.01	-0.04	[-0.05-(-0.04)]	<0.01
BMI, (kg/m^2)		<0.01	[<0.01-<0.01]	<0.01	<0.01	[<0.01-<0.01]	<0.01	<0.01	[<0.01-<0.01]	<0.01
Pre-operative ASA physical function score (Mild disease not incapacitating, grade 2)	Fit and healthy (grade 1)	-0.06	[-0.07-(-0.05)]	<0.01	-0.05	[-0.05-(-0.04)]	<0.01	-0.03	[-0.04-(-0.03)]	<0.01

© 2019 Garriga C et al. *JAMA Network Open.*

	Incapacitating systemic disease (grade 3)	0.20	[0.19-0.20]	<0.01	0.18	[0.18-0.19]	<0.01	0.15	[0.15-0.16]	<0.01
	Life threatening disease or Expected to die within 24h (grades 4 and 5)	0.39	[0.36-0.42]	<0.01	0.37	[0.34-0.40]	<0.01	0.32	[0.29-0.35]	<0.01
IMD (Most deprived 20%)	Least deprived 20%	-0.03	[-0.04-(-0.02)]	<0.01	-0.02	[-0.03-(-0.01)]	<0.01	-0.01	[-0.02-<0.01]	0.03
	Less deprived 20-40%	-0.01	[-0.02-<0.01]	0.02	-0.01	[-0.02-<0.01]	0.16	<0.01	[-0.01-0.01]	0.99
	Less deprived 40-60%	-0.01	[-0.02-<0.01]	0.22	<0.01	[-0.01-0.01]	0.38	<0.01	[-0.01-0.01]	0.52
	More deprived 20-40%	<0.01	[-0.01-0.01]	0.85	<0.01	[-0.01-0.01]	0.94	<0.01	[-0.01-0.01]	0.79
Rural/Urban indicator (Urban >=10,000 habitants)	Town and fringe	-0.01	[-0.02-<0.01]	0.20	-0.01	[-0.02-<0.01]	0.19	<0.01	[-0.01-0.01]	0.40

© 2019 Garriga C et al. *JAMA Network Open.*

	Village/isolated	-0.02	[-0.03-(-0.01)]	<0.01	-0.02	[-0.03-(-0.01)]	<0.01	-0.01	[-0.02-<0.01]	0.02
Primary indication (Osteoarthritis)	Osteoarthritis and Other*	0.16	[0.15-0.18]	<0.01	0.14	[0.12-0.16]	<0.01	0.11	[0.09-0.13]	<0.01
Charlson score (None, 0)	Mild (1)	0.07	[0.07-0.08]	<0.01	0.08	[0.07-0.08]	<0.01	0.07	[0.06-0.07]	<0.01
	Moderate (2)	0.18	[0.17-0.19]	<0.01	0.18	[0.17-0.18]	<0.01	0.16	[0.15-0.16]	<0.01
	Severe (≥3)	0.31	[0.30-0.32]	<0.01	0.31	[0.30-0.32]	<0.01	0.28	[0.27-0.29]	<0.01
EQ-5D-3L (Lowest quintile)	2nd quintile	-0.10	[-0.11-(-0.10)]	<0.01	-0.10	[-0.11-(-0.09)]	<0.01	-0.10	[-0.10-(-0.09)]	<0.01
	3rd quintile	-0.13	[-0.14-(-0.13)]	<0.01	-0.13	[-0.14-(-0.12)]	<0.01	-0.12	[-0.13-(-0.12)]	<0.01
	4th quintile	-0.16	[-0.17-(-0.15)]	<0.01	-0.15	[-0.16-(-0.14)]	<0.01	-0.14	[-0.15-(-0.13)]	<0.01
	Highest quintile	-0.20	[-0.21-(-0.19)]	<0.01	-0.19	[-0.20-(-0.19)]	<0.01	-0.18	[-0.19-(-0.18)]	<0.01
Surgical factors										
Lead surgeon experience (Consultant)	Other**				0.09	[0.08-0.09]	<0.01	0.01	[<0.01-0.02]	<0.01
Surgical volume per lead surgeon (>150 surgeries per year)	≤10				0.10	[0.08-0.12]	<0.01	0.02	[0.01-0.04]	0.01

© 2019 Garriga C et al. *JAMA Network Open.*

	11-50				0.08	[0.07-0.08]	<0.01	0.01	[0.01-0.02]	<0.01
	51-75				0.07	[0.06-0.08]	<0.01	0.02	[0.01-0.03]	<0.01
	76-100				0.05	[0.05-0.06]	<0.01	0.01	[<0.01-0.02]	<0.01
	101-150				0.03	[0.02-0.04]	<0.01	<0.01	[-0.01-0.01]	0.69
Surgical volume per unit (Surgeries per year, ≥500)	≤200				0.06	[0.05-0.07]	<0.01	0.10	[0.09-0.11]	<0.01
	200-299				0.01	[<0.01-0.01]	0.24	0.01	[<0.01-0.02]	<0.01
	300-399				-0.03	[-0.04-(-0.02)]	<0.01	-0.02	[-0.03-(-0.02)]	<0.01
	400-499				-0.03	[-0.04-(-0.02)]	<0.01	-0.02	[-0.03-(-0.01)]	<0.01
Minimally invasive surgery (No)	Yes				-0.05	[-0.07-(-0.04)]	<0.01	-0.04	[-0.05-(-0.03)]	<0.01
Thromboprophylaxis (LMWH +/-Other)	None				-0.05	[-0.07-(-0.03)]	<0.01	-0.02	[-0.04-0.01]	0.19
	Aspirin only				-0.02	[-0.04-(-0.01)]	0.01	-0.02	[-0.03-<0.01]	0.01
	Other (no LMWH)				-0.01	[-0.02-(-0.01)]	<0.01	0.01	[<0.01-0.02]	<0.01
Mechanical prophylaxis (any)	None				0.08	[0.06-0.09]	<0.01	0.02	[<0.01-0.03]	0.02

© 2019 Garriga C et al. *JAMA Network Open.*

General anaesthesia (No)	Yes				0.05	[0.05-0.06]	<0.01	0.05	[0.05-0.06]	<0.01
Regional – epidural anaesthesia (No)	Yes				0.04	[0.03-0.05]	<0.01	0.02	[0.01-0.03]	<0.01
Regional – nerve block anaesthesia (No)	Yes				0.04	[0.04-0.05]	<0.01	0.03	[0.02-0.04]	<0.01
Regional – spinal anaesthesia (No)	Yes									
Type of approach (Medial parapatellar)	Lateral parapatellar				0.09	[0.06-0.11]	<0.01	0.07	[0.05-0.09]	<0.01
	Mid-Vastus				-0.06	[-0.07-(-0.05)]	<0.01	-0.07	[-0.08-(-0.05)]	<0.01
	Sub-Vastus				-0.05	[-0.07-(-0.02)]	<0.01	-0.03	[-0.05-(-0.01)]	0.01
	Other				0.04	[0.02-0.06]	<0.01	0.04	[0.02-0.06]	<0.01
Femoral bone graft (No)	Yes				0.02	[<0.01-0.04]	0.04	0.03	[<0.01-0.05]	0.02
Tibia bone graft (No)	Yes				0.10	[0.07-0.14]	<0.01	0.09	[0.05-0.12]	<0.01
Primary femoral fixation (Cemented)	Cementless									

© 2019 Garriga C et al. *JAMA Network Open.*

Primary Tibial fixation (Cemented)	Cementless									
Type of knee implant (TKR)	UKR				0.26	[0.25-0.28]	<0.01	0.28	[0.27-0.29]	<0.01
Hospital organisation factors										
Unit type (Public hospital)	Private hospital							-0.26	[-0.27-(-0.26)]	<0.01
	Independent sector (private) – treatment centre							-0.44	[-0.45-(-0.42)]	<0.01
FTE of speciality groups on trauma and orthopaedic surgery (0-24 FTEs)	25-29							0.02	[0.01-0.03]	<0.01
	30-39							0.02	[0.02-0.03]	<0.01
	40-49							0.05	[0.04-0.06]	<0.01
	>50							0.06	[0.05-0.07]	<0.01

© 2019 Garriga C et al. *JAMA Network Open.*

Consultant FTE (0-24 FTEs)	25-29							0.02	[0.01-0.03]	<0.01
	30-39							0.01	[<0.01-0.02]	0.02
	40-49							0.02	[0.01-0.03]	<0.01
	>50							0.04	[0.02-0.05]	<0.01
middle grade doctor FTE (0-24 FTEs)	25-29							0.01	[<0.01-0.02]	0.01
	30-39							0.04	[0.03-0.05]	<0.01
	40-49							0.03	[0.02-0.05]	<0.01
	>50							0.04	[0.02-0.06]	<0.01
early career doctor FTE (0-24 FTEs)	25-29									
	30-39									
Total beds available overnight (0-349)	350-499							-0.03	[-0.04-(-0.02)]	<0.01
	500-699							-0.06	[-0.07-(-0.05)]	<0.01
	700-999							-0.08	[-0.09-(-0.06)]	<0.01
	≥1000							-0.10	[-0.12-(-0.08)]	<0.01

© 2019 Garriga C et al. *JAMA Network Open.*

Total beds available overnight for trauma and orthopaedic surgery (0-34)	35-49							0.02	[0.01-0.03]	<0.01
	50-69							0.03	[0.03-0.04]	<0.01
	70-99							0.04	[0.02-0.05]	<0.01
	≥100							0.05	[0.03-0.07]	<0.01
Total beds available overnight for rehabilitation (0)	>0-10							<0.01	[-0.02-0.01]	0.92
	11-20							<0.01	[-0.02-0.01]	0.60
	≥20							0.03	[0.02-0.04]	<0.01
Operating theatres (<10)	10-14							-0.02	[-0.02-(-0.01)]	<0.01
	15-19							-0.01	[-0.02-<0.01]	0.19
	20-24							-0.04	[-0.06-(-0.03)]	<0.01
	≥25							-0.06	[-0.08-(-0.04)]	<0.01
Dedicated day case operating theatres (≥7)	0							<0.01	[-0.01-0.01]	0.37

© 2019 Garriga C et al. *JAMA Network Open.*

	1-2							0.01	[<0.01-0.01]	0.22
	3-4							<0.01	[-0.01-0.01]	0.51
	5-6							0.01	[<0.01-0.02]	0.01

Total knee replacement, TKR; Unicompartmental knee replacement, UKR; American Society of Anesthesiologists, ASA; Index of multiple deprivation, IMD; European Quality of life-five domain, EQ-5D-3L; low-molecular-weight heparin, LMWH; full time equivalent, FTE (proportion of full time contracted hours).

© 2019 Garriga C et al. *JAMA Network Open.*

eTable 4. Correlation Coefficients (ρ) of Hospital Organizational Factors vs Predicted Outcomes of Surgery for Patients Undergoing Primary Joint Replacement, 2014-2016, England, United Kingdom

	**primary hip replacement**				**primary TKR/UKR**			
**Mean values by centre and health area**	**LOS**	**Bed-day costs**	**OHS**	**Complications**	**LOS**	**Bed-day costs**	**OKS**	**Complications**
Surgical volume per lead surgeon (number of surgeries per year)	**-0.36**	**-0.34**	0.03	**-0.40**	**-0.40**	**-0.39**	0.11	**-0.37**
Surgical volume per unit (number of surgeries per year)	-0.05	-0.04	-0.04	**-0.38**	0.05	0.05	-0.01	-0.26
Public hospital (%)	**0.41**	**0.40**	-0.14	0.22	**0.44**	**0.43**	-0.19	0.27
Private hospital (%)	**-0.37**	**-0.35**	0.07	-0.27	**-0.38**	**-0.35**	0.12	-0.25
ISTC (%)	-0.15	-0.17	0.15	0.04	-0.19	-0.21	0.15	-0.09
FTE of speciality groups on trauma and orthopaedic surgery	0.11	0.10	-0.06	-0.08	0.10	0.11	0.02	0.05
consultants FTE	0.12	0.09	-0.12	0.09	0.14	0.13	-0.06	0.14
middle grade doctor FTE	0.26	0.23	-0.18	0.20	0.20	0.21	-0.11	0.19
early career doctor FTE	0.27	0.27	-0.08	0.21	0.10	0.08	-0.04	0.19
Total beds available overnight	0.05	0.01	-0.06	0.09	0.01	0.01	0.03	0.12
Total beds available overnight for trauma and orthopaedic surgery	-0.03	-0.04	-0.003	-0.18	-0.08	-0.07	0.16	-0.05
Total beds available overnight for rehabilitation	0.10	0.09	-0.04	-0.0003	0.02	0.03	0.07	-0.09
Operating theatres	0.08	0.05	-0.04	0.09	0.10	0.10	0.01	0.11
Dedicated day case operating theatres	-0.04	-0.04	-0.003	0.06	0.01	0.02	-0.003	-0.05

LOS, length of stay; OHS, Oxford hip score; OKS, Oxford knee score; FTE (proportion of full time contracted hours); ISTC, independent sector treatment centre; TKR, total knee replacement; UKR, unicompartmental knee replacement.

© 2019 Garriga C et al. *JAMA Network Open.*

eTable 5. Poisson Multilevel Models of Bed-Day Costs for Patients Who Underwent Planned Primary Hip Replacement

		Patient model			Surgical model			Hospital organisation model		
	Categories	Coef.	95% CI	P	Coef.	95% CI	P	Coef.	95% CI	P
Intercept		8.68	[8.67-8.70]	<0.01	8.67	[8.65-8.68]	<0.01	8.66	[8.65-8.67]	<0.01
Patient factors										
Calendar year (2016)	2015	0.08	[0.08-0.08]	<0.01	0.07	[0.07-0.07]	<0.01	0.06	[0.06-0.06]	<0.01
	2016	0.04	[0.04-0.04]	<0.01	0.03	[0.03-0.03]	<0.01	0.03	[0.03-0.03]	<0.01
Age (<50 years)	50-59	<0.01	[<0.01-<0.01]	<0.01	<0.01	[<0.01-<0.01]	<0.01	0.01	[0.01-0.01]	<0.01
	60-69	0.02	[0.02-0.02]	<0.01	0.01	[0.01-0.01]	<0.01	0.03	[0.03-0.03]	<0.01
	70-79	0.12	[0.12-0.12]	<0.01	0.10	[0.09-0.10]	<0.01	0.11	[0.11-0.11]	<0.01
	80-84	0.28	[0.28-0.28]	<0.01	0.25	[0.25-0.25]	<0.01	0.26	[0.26-0.26]	<0.01
	≥85	0.44	[0.44-0.44]	<0.01	0.41	[0.41-0.41]	<0.01	0.42	[0.42-0.42]	<0.01
Sex (woman)	man	-0.09	[-0.09-(-0.09)]	<0.01	-0.09	[-0.09-(-0.09)]	<0.01	-0.09	[-0.09-(-0.09)]	<0.01
BMI, (kg/m^2)		<0.01	[<0.01-<0.01]	<0.01	<0.01	[<0.01-<0.01]	<0.01	<0.01	[<0.01-<0.01]	0.01
Pre-operative ASA physical function score (Mild disease not incapacitating, grade 2)	Fit and healthy (grade 1)	-0.02	[-0.02-(-0.02)]	<0.01	-0.02	[-0.02-(-0.02)]	<0.01	-0.02	[-0.02-(-0.02)]	<0.01

© 2019 Garriga C et al. *JAMA Network Open.*

	Incapacitating systemic disease (grade 3)	0.12	[0.12-0.12]	<0.01	0.11	[0.11-0.12]	<0.01	0.09	[0.09-0.09]	<0.01
	Life threatening disease or Expected to die within 24h (grades 4 and 5)	0.23	[0.23-0.23]	<0.01	0.21	[0.21-0.22]	<0.01	0.18	[0.18-0.18]	<0.01
IMD (Most deprived 20%)	Least deprived 20%	-0.03	[-0.04-(-0.02)]	<0.01	-0.02	[-0.03-(-0.01)]	<0.01	-0.02	[-0.03-(-0.01)]	<0.01
	Less deprived 20-40%	-0.02	[-0.03-(-0.01)]	<0.01	-0.02	[-0.03-(-0.01)]	<0.01	-0.01	[-0.02-<0.01)]	0.01
	Less deprived 40-60%	-0.01	[-0.02-<0.01]	0.07	-0.01	[-0.02-<0.01]	0.05	-0.01	[-0.02-<0.01]	0.04
	More deprived 20-40%	-0.01	[-0.02-<0.01]	0.08	-0.01	[-0.02-<0.01]	0.08	-0.01	[-0.02-<0.01]	0.08
Rural/Urban indicator (Urban >=10,000 habitants)	Town and fringe	0.01	[0.01-0.01]	<0.01	0.01	[0.01-0.01]	<0.01	0.01	[0.01-0.01]	<0.01

© 2019 Garriga C et al. *JAMA Network Open.*

	Village/isolated	0.01	[0.01-0.01]	<0.01	0.01	[0.01-0.01]	<0.01	0.01	[<0.01-0.01]	<0.01
Primary indication (Osteoarthritis)	Osteoarthritis and Other*	0.12	[0.12-0.12]	<0.01	0.11	[0.11-0.12]	<0.01	0.09	[0.09-0.09]	<0.01
Charlson score (None, 0)	Mild (1)	0.01	[0.01-0.01]	<0.01	0.01	[0.01-0.01]	<0.01	0.01	[0.01-0.01]	<0.01
	Moderate (2)	0.05	[0.05-0.05]	<0.01	0.05	[0.05-0.05]	<0.01	0.04	[0.03-0.04]	<0.01
	Severe (≥3)	0.10	[0.10-0.10]	<0.01	0.09	[0.09-0.09]	<0.01	0.08	[0.08-0.08]	<0.01
EQ-5D-3L (Highest quintile)	Lowest quintile	-0.09	[-0.09-(-0.09)]	<0.01	-0.09	[-0.09-(-0.09)]	<0.01	-0.09	[-0.09-(-0.09)]	<0.01
	2nd quintile	-0.12	[-0.12-(-0.12)]	<0.01	-0.11	[-0.11-(-0.11)]	<0.01	-0.11	[-0.11-(-0.11)]	<0.01
	3rd quintile	-0.14	[-0.14-(-0.14)]	<0.01	-0.13	[-0.13-(-0.13)]	<0.01	-0.13	[-0.13-(-0.13)]	<0.01
	4th quintile	-0.16	[-0.16-(-0.16)]	<0.01	-0.16	[-0.16-(-0.16)]	<0.01	-0.15	[-0.15-(-0.15)]	<0.01
Surgical factors										
Lead surgeon experience (Consultant)	Other**				0.07	[0.07-0.07]	<0.01	0.02	[0.02-0.02]	<0.01
Surgical volume per lead surgeon (>150 surgeries per year)	≤10				0.07	[0.07-0.07]	<0.01	0.02	[0.02-0.02]	<0.01

© 2019 Garriga C et al. *JAMA Network Open.*

	11-50				0.06	[0.06-0.06]	<0.01	0.02	[0.02-0.02]	<0.01
	51-75				0.03	[0.03-0.03]	<0.01	0.01	[0.01-0.01]	<0.01
	76-100				0.02	[0.02-0.02]	<0.01	<0.01	[<0.01-<0.01]	0.02
	101-150				0.02	[0.02-0.02]	<0.01	0.01	[0.01-0.01]	<0.01
Surgical volume per unit (Surgeries per year, ≥500)	≤200				0.05	[0.05-0.05]	<0.01	0.11	[0.11-0.11]	<0.01
	200-299				0.01	[0.01-0.01]	<0.01	0.05	[0.05-0.06]	<0.01
	300-399				-0.02	[-0.02-(-0.02)]	<0.01	0.02	[0.02-0.02]	<0.01
	400-499				-0.01	[-0.01-(-0.01)]	<0.01	0.05	[0.05-0.05]	<0.01
Minimally invasive surgery (No)	Yes				-0.04	[-0.04-(-0.04)]	<0.01	-0.03	[-0.03-(-0.03)]	<0.01
Thromboprophylaxis (LMWH +/-Other)	None				<0.01	[<0.01-<0.01]	<0.01	0.02	[0.02-0.02]	<0.01
	Aspirin only				-0.02	[-0.02-(-0.02)]	<0.01	-0.01	[-0.01-(-0.01)]	<0.01
	Other (no LMWH)				-0.02	[-0.02-(-0.02)]	<0.01	<0.01	[<0.01-<0.01]	<0.01
Mechanical prophylaxis (any)	None				0.05	[0.05-0.05]	<0.01	0.01	[0.01-0.01]	<0.01

© 2019 Garriga C et al. *JAMA Network Open.*

General anaesthesia (No)	Yes				0.04	[0.03-0.04]	<0.01	0.03	[0.03-0.03]	<0.01
Regional – epidural anaesthesia (No)	Yes				0.01	[0.01-0.01]	<0.01	<0.01	[<0.01-<0.01]	<0.01
Regional – nerve block anaesthesia (No)	Yes				0.04	[0.04-0.04]	<0.01	0.03	[0.03-0.03]	<0.01
Regional – spinal anaesthesia (No)	Yes				-0.02	[-0.02-(-0.02)]	<0.01	-0.02	[-0.02-(-0.02)]	<0.01
Type of approach (Posterior)	Anterior, antero-lateral, hardinge, lateral, trochanteric osteotomy, other				0.05	[0.05-0.05]	<0.01	0.05	[0.05-0.05]	<0.01
Femoral bone graft (No)	Yes				0.04	[0.04-0.04]	<0.01	0.04	[0.04-0.05]	<0.01
Cup bone graft (No)	Yes				0.05	[0.05-0.05]	<0.01	0.04	[0.04-0.04]	<0.01

© 2019 Garriga C et al. *JAMA Network Open.*

Primary cup fixation (Cementless)	Cemented				-0.01	[-0.01-(-0.01)]	<0.01	-0.01	[-0.01-(-0.01)]	<0.01
Type of primary stem fixation (Cemented THR stem)	Not available or Resurfacing				-0.01	[-0.01-(-0.01)]	<0.01	<0.01	[<0.01-0.01]	<0.01
	Cementless THR stem				-0.05	[-0.05-(-0.05)]	<0.01	-0.03	[-0.03-(-0.03)]	<0.01
Bearing surface (MoP)	MoM				-0.03	[-0.03-(-0.03)]	<0.01	-0.08	[-0.08-(-0.07)]	<0.01
	CoC				<0.01	[<0.01-<0.01]	<0.01	-0.02	[-0.02-(-0.02)]	<0.01
	CoP				-0.01	[-0.01-(-0.01)]	<0.01	-0.02	[-0.02-(-0.02)]	<0.01
	CoM or MoC or unknown				0.07	[0.07-0.07]	<0.01	0.05	[0.05-0.05]	<0.01
Femoral head size (≤28mm)	32				<0.01	[<0.01-<0.01]	<0.01	<0.01	[<0.01-<0.01]	<0.01
	36-42				0.02	[0.02-0.02]	<0.01	0.02	[0.02-0.02]	<0.01
	≥44				0.02	[0.01-0.02]	<0.01	0.01	[<0.01-0.01]	<0.01
Hospital organisation factors											

© 2019 Garriga C et al. *JAMA Network Open.*

Unit type (Public hospital)	Private hospital							-0.15	[-0.15-(-0.14)]	<0.01
	Independent sector (private) – treatment centre							-0.29	[-0.29-(-0.29)]	<0.01
FTE of speciality groups on trauma and orthopaedic surgery (0-24 FTEs)	25-29							<0.01	[<0.01-<0.01]	<0.01
	30-39							0.02	[0.02-0.02]	<0.01
	40-49							0.01	[0.01-0.01]	<0.01
	>50							0.03	[0.03-0.03]	<0.01
Consultant FTE (0-24 FTEs)	25-29							0.01	[0.01-0.01]	<0.01
	30-39							0.03	[0.03-0.03]	<0.01
	40-49							0.02	[0.02-0.02]	<0.01
	>50							0.03	[0.03-0.03]	<0.01

© 2019 Garriga C et al. *JAMA Network Open.*

middle grade doctor FTE (0-24 FTEs)	25-29							0.01	[0.01-0.01]	<0.01
	30-39							<0.01	[<0.01-<0.01]	<0.01
	40-49							0.01	[0.01-0.01]	<0.01
	>50							0.02	[0.02-0.02]	<0.01
early career doctor FTE (0-24 FTEs)	25-29							<0.01	[<0.01-<0.01]	<0.01
	30-39							-0.05	[-0.05-(-0.05)]	<0.01
Total beds available overnight (0-349)	350-499							-0.03	[-0.03-(-0.03)]	<0.01
	500-699							-0.03	[-0.03-(-0.03)]	<0.01
	700-999							-0.04	[-0.04-(-0.04)]	<0.01
	≥1000							-0.07	[-0.07-(-0.07)]	<0.01
Total beds available overnight for trauma and orthopaedic surgery (0-34)	35-49							0.02	[0.02-0.02]	<0.01
	50-69							0.05	[0.05-0.05]	<0.01
	70-99							0.08	[0.08-0.08]	<0.01

© 2019 Garriga C et al. *JAMA Network Open.*

	≥100							0.12	[0.12-0.12]	<0.01
Total beds available overnight for rehabilitation (0)	>0-10							<0.01	[<0.01-<0.01]	<0.01
	11-20							0.01	[0.01-0.01]	<0.01
	≥20							0.06	[0.06-0.06]	<0.01
Operating theatres (<10)	10-14							0.01	[0.01-0.01]	<0.01
	15-19							-0.02	[-0.02-(-0.02)]	<0.01
	20-24							-0.06	[-0.06-(-0.06)]	<0.01
	≥25							-0.05	[-0.05-(-0.05)]	<0.01
Dedicated day case operating theatres (≥7)	0							0.05	[0.05-0.05]	<0.01
	1-2							0.04	[0.04-0.04]	<0.01
	3-4							0.04	[0.04-0.04]	<0.01
	5-6							0.02	[0.02-0.02]	<0.01

American Society of Anesthesiologists, ASA; Index of multiple deprivation, IMD; European Quality of life-five domain, EQ-5D-3L; low-molecular-weight heparin, LMWH; total hip replacement, THR; metal-on-metal, MoM; metal-on-polyethylene, MoP; ceramic-on-ceramic, CoC; ceramic-on-polyethylene, CoP; ceramic-on-metal, CoM; metal-on-ceramic, MoC; full time equivalent, FTE (proportion of full time contracted hours).

© 2019 Garriga C et al. *JAMA Network Open.*

eTable 6. Poisson Multilevel Models of Bed-Day Costs for Patients Who Underwent Planned Primary TKR/UKR

		**Patient model**			**Surgical model**			**Hospital organisation model**		
	Categories	Coef.	95% CI	P	Coef.	95% CI	P	Coef.	95% CI	P
Intercept		8.55	[8.53-8.57]	<0.01	8.35	[8.34-8.37]	<0.01	8.47	[8.46-8.49]	<0.01
Patient factors										
Calendar year (2016)	2015	0.07	[0.07-0.07]	<0.01	0.06	[0.06-0.06]	<0.01	0.06	[0.06-0.06]	<0.01
	2016	0.03	[0.03-0.04]	<0.01	0.03	[0.03-0.03]	<0.01	0.03	[0.03-0.03]	<0.01
Age (<50 years)	50-59	-0.02	[-0.02-(-0.01)]	<0.01	-0.03	[-0.03-(-0.03)]	<0.01	-0.03	[-0.03-(-0.03)]	<0.01
	60-69	0.02	[0.02-0.02]	<0.01	-0.02	[-0.02-(-0.02)]	<0.01	-0.01	[-0.01-(-0.01)]	<0.01
	70-79	0.10	[0.10-0.10]	<0.01	0.06	[0.06-0.06]	<0.01	0.07	[0.07-0.07]	<0.01
	80-84	0.26	[0.26-0.26]	<0.01	0.20	[0.20-0.21]	<0.01	0.21	[0.21-0.21]	<0.01
	≥85	0.41	[0.41-0.41]	<0.01	0.35	[0.35-0.35]	<0.01	0.35	[0.35-0.35]	<0.01
Sex (woman)	man	-0.06	[-0.06-(-0.06)]	<0.01	-0.05	[-0.05-(-0.05)]	<0.01	-0.05	[-0.05-(-0.05)]	<0.01
BMI, (kg/m^2)		<0.01	[<0.01-<0.01]	<0.01	<0.01	[<0.01-<0.01]	<0.01	<0.01	[<0.01-<0.01]	<0.01
Pre-operative ASA physical function score (Mild disease not incapacitating, grade 2)	Fit and healthy (grade 1)	-0.03	[-0.03-(-0.03)]	<0.01	-0.02	[-0.02-(-0.02)]	<0.01	-0.01	[-0.01-(-0.01)]	<0.01

© 2019 Garriga C et al. *JAMA Network Open.*

	Incapacitating systemic disease (grade 3)	0.12	[0.12-0.12]	<0.01	0.11	[0.11-0.11]	<0.01	0.09	[0.09-0.09]	<0.01
	Life threatening disease or Expected to die within 24h (grades 4 and 5)	0.23	[0.23-0.23]	<0.01	0.20	[0.20-0.20]	<0.01	0.17	[0.17-0.17]	<0.01
IMD (Most deprived 20%)	Least deprived 20%	-0.02	[-0.04-(-0.01)]	<0.01	-0.02	[-0.03-(-0.01)]	<0.01	-0.01	[-0.02-<0.01]	0.01
	Less deprived 20-40%	-0.01	[-0.02-<0.01]	0.01	-0.01	[-0.02-<0.01]	0.05	<0.01	[-0.01-0.01]	0.45
	Less deprived 40-60%	<0.01	[-0.01-0.01]	0.35	<0.01	[-0.01-0.01]	0.58	<0.01	[-0.01-0.01]	0.73
	More deprived 20-40%	<0.01	[-0.01-0.01]	0.74	<0.01	[-0.01-0.01]	0.91	<0.01	[-0.01-0.01]	0.99
Rural/Urban indicator (Urban >=10,000 habitants)	Town and fringe	0.02	[0.02-0.02]	<0.01	0.01	[0.01-0.01]	<0.01	0.01	[0.01-0.01]	<0.01

© 2019 Garriga C et al. *JAMA Network Open.*

	Village/isolated	<0.01	[<0.01-<0.01]	<0.01	<0.01	[<0.01-<0.01]	<0.01	<0.01	[<0.01-<0.01]	<0.01
Primary indication (Osteoarthritis)	Osteoarthritis and Other*	0.14	[0.14-0.14]	<0.01	0.11	[0.11-0.11]	<0.01	0.08	[0.08-0.08]	<0.01
Charlson score (None, 0)	Mild (1)	0.03	[0.03-0.03]	<0.01	0.03	[0.03-0.03]	<0.01	0.02	[0.02-0.02]	<0.01
	Moderate (2)	0.07	[0.07-0.07]	<0.01	0.07	[0.07-0.07]	<0.01	0.05	[0.05-0.05]	<0.01
	Severe (≥3)	0.11	[0.11-0.12]	<0.01	0.11	[0.11-0.11]	<0.01	0.09	[0.09-0.09]	<0.01
EQ-5D-3L (Lowest quintile)	2nd quintile	-0.07	[-0.07-(-0.07)]	<0.01	-0.07	[-0.07-(-0.07)]	<0.01	-0.07	[-0.07-(-0.07)]	<0.01
	3rd quintile	-0.10	[-0.10-(-0.10)]	<0.01	-0.09	[-0.09-(-0.09)]	<0.01	-0.08	[-0.08-(-0.08)]	<0.01
	4th quintile	-0.11	[-0.11-(-0.11)]	<0.01	-0.11	[-0.11-(-0.11)]	<0.01	-0.10	[-0.10-(-0.10)]	<0.01
	Highest quintile	-0.14	[-0.14-(-0.14)]	<0.01	-0.13	[-0.14-(-0.13)]	<0.01	-0.13	[-0.13-(-0.13)]	<0.01
Surgical factors										
Lead surgeon experience (Consultant)	Other**				0.07	[0.07-0.07]	<0.01	0.01	[0.01-0.01]	<0.01
Surgical volume per lead surgeon (>150 surgeries per year)	≤10				0.08	[0.08-0.08]	<0.01	0.02	[0.02-0.02]	<0.01

© 2019 Garriga C et al. *JAMA Network Open.*

	11-50				0.06	[0.06-0.06]	<0.01	0.01	[0.01-0.01]	<0.01
	51-75				0.05	[0.05-0.06]	<0.01	0.02	[0.02-0.02]	<0.01
	76-100				0.04	[0.04-0.04]	<0.01	0.01	[0.01-0.01]	<0.01
	101-150				0.02	[0.02-0.02]	<0.01	<0.01	[<0.01-<0.01]	<0.01
Surgical volume per unit (Surgeries per year, ≥500)	≤200				0.05	[0.05-0.05]	<0.01	0.08	[0.08-0.08]	<0.01
	200-299				0.01	[0.01-0.01]	<0.01	0.01	[0.01-0.01]	<0.01
	300-399				-0.02	[-0.02-(-0.02)]	<0.01	-0.02	[-0.02-(-0.02)]	<0.01
	400-499				-0.02	[-0.02-(-0.02)]	<0.01	-0.02	[-0.02-(-0.02)]	<0.01
Minimally invasive surgery (No)	Yes				-0.03	[-0.03-(-0.03)]	<0.01	-0.02	[-0.02-(-0.02)]	<0.01
Thromboprophylaxis (LMWH +/-Other)	None				-0.03	[-0.03-(-0.03)]	<0.01	-0.01	[-0.01-<0.01]	<0.01
	Aspirin only				-0.01	[-0.01-(-0.01)]	<0.01	-0.01	[-0.01-<0.01]	<0.01
	Other (no LMWH)				-0.01	[-0.01-<0.01]	<0.01	0.01	[0.01-0.01]	<0.01
Mechanical prophylaxis (any)	None				0.07	[0.07-0.07]	<0.01	0.02	[0.02-0.02]	<0.01

© 2019 Garriga C et al. *JAMA Network Open.*

General anaesthesia (No)	Yes				0.04	[0.04-0.04]	<0.01	0.04	[0.04-0.04]	<0.01
Regional – epidural anaesthesia (No)	Yes				0.03	[0.03-0.03]	<0.01	0.02	[0.02-0.02]	<0.01
Regional – nerve block anaesthesia (No)	Yes				0.04	[0.03-0.04]	<0.01	0.02	[0.02-0.02]	<0.01
Regional – spinal anaesthesia (No)	Yes				-0.01	[-0.01-(-0.01)]	<0.01	<0.01	[<0.01-<0.01]	<0.01
Type of approach (Medial parapatellar)	Lateral parapatellar				0.06	[0.05-0.06]	<0.01	0.04	[0.04-0.04]	<0.01
	Mid-Vastus				-0.04	[-0.04-(-0.04)]	<0.01	-0.05	[-0.05-(-0.05)]	<0.01
	Sub-Vastus				-0.05	[-0.05-(-0.04)]	<0.01	-0.03	[-0.03-(-0.03)]	<0.01
	Other				0.02	[0.02-0.03]	<0.01	0.03	[0.02-0.03]	<0.01
Femoral bone graft (No)	Yes				0.02	[0.02-0.02]	<0.01	0.03	[0.03-0.03]	<0.01
Tibia bone graft (No)	Yes				0.07	[0.07-0.07]	<0.01	0.06	[0.06-0.06]	<0.01
Primary femoral fixation (Cemented)	Cementless				0.03	[0.03-0.03]	<0.01	0.03	[0.03-0.04]	<0.01

© 2019 Garriga C et al. *JAMA Network Open.*

Primary Tibial fixation (Cemented)	Cementless				-0.02	[-0.02-(-0.02)]	<0.01	-0.03	[-0.03-(-0.03)]	<0.01
Type of knee implant (TKR)	UKR				0.22	[0.22-0.22]	<0.01	0.22	[0.22-0.22]	<0.01
Hospital organisation factors										
Unit type (Public hospital)	Private hospital							-0.19	[-0.19-(-0.19)]	<0.01
	Independent sector (private) – treatment centre							-0.33	[-0.33-(-0.33)]	<0.01
FTE of speciality groups on trauma and orthopaedic surgery (0-24 FTEs)	25-29							0.02	[0.02-0.02]	<0.01
	30-39							0.02	[0.02-0.02]	<0.01
	40-49							0.03	[0.03-0.03]	<0.01
	>50							0.04	[0.04-0.04]	<0.01

© 2019 Garriga C et al. *JAMA Network Open.*

Consultant FTE (0-24 FTEs)	25-29							0.01	[0.01-0.01]	<0.01
	30-39							0.01	[0.01-0.01]	<0.01
	40-49							0.01	[0.01-0.01]	<0.01
	>50							0.03	[0.03-0.03]	<0.01
middle grade doctor FTE (0-24 FTEs)	25-29							0.01	[0.01-0.01]	<0.01
	30-39							0.03	[0.02-0.03]	<0.01
	40-49							0.03	[0.03-0.03]	<0.01
	>50							0.05	[0.04-0.05]	<0.01
early career doctor FTE (0-24 FTEs)	25-29							<0.01	[<0.01-<0.01]	<0.01
	30-39							0.03	[0.03-0.03]	<0.01
Total beds available overnight (0-349)	350-499							-0.02	[-0.02-(-0.02]	<0.01
	500-699							-0.04	[-0.05-(-0.04]	<0.01
	700-999							-0.06	[-0.06-(-0.06]	<0.01
	≥1000							-0.07	[-0.07-(-0.07]	<0.01

© 2019 Garriga C et al. *JAMA Network Open.*

Total beds available overnight for trauma and orthopaedic surgery (0-34)	35-49							0.01	[0.01-0.01]	<0.01
	50-69							0.02	[0.02-0.02]	<0.01
	70-99							0.03	[0.03-0.03]	<0.01
	≥100							0.04	[0.04-0.04]	<0.01
Total beds available overnight for rehabilitation (0)	>0-10							<0.01	[<0.01-<0.01]	<0.01
	11-20							<0.01	[<0.01-<0.01]	<0.01
	≥20							0.02	[0.02-0.02]	<0.01
Operating theatres (<10)	10-14							-0.01	[-0.01-(-0.01)]	<0.01
	15-19							-0.01	[-0.01-(-0.01)]	<0.01
	20-24							-0.03	[-0.03-(-0.03)]	<0.01
	≥25							-0.05	[-0.05-(-0.05)]	<0.01
Dedicated day case operating theatres (≥7)	0							<0.01	[<0.01-<0.01]	<0.01

© 2019 Garriga C et al. *JAMA Network Open.*

	1-2							<0.01	[<0.01-<0.01]	<0.01
	3-4							<0.01	[<0.01-<0.01]	<0.01
	5-6							0.02	[0.02-0.02]	<0.01

Total knee replacement, TKR; Unicompartmental knee replacement, UKR; American Society of Anesthesiologists, ASA; Index of multiple deprivation, IMD; European Quality of life-five domain, EQ-5D-3L; low-molecular-weight heparin, LMWH; full time equivalent, FTE (proportion of full time contracted hours).

© 2019 Garriga C et al. *JAMA Network Open.*

eTable 7. Continuous Multilevel Models of Oxford Hip Score for Patients Who Underwent Planned Primary Hip Replacement

		Patient model			Surgical model			Hospital organisation model		
	Categories	Coef.	95% CI	P	Coef.	95% CI	P	Coef.	95% CI	P
Intercept		35.1	[34.3-35.8]	<0.01	36.3	[35.4-37.1]	<0.01	35.9	[35.1-36.8]	<0.01
Patient factors										
Calendar year (2016)	2015	-0.4	[-0.5-(-0.2)]	<0.01	-0.2	[-0.4-(-0.1)]	0.01	-0.2	[-0.4-(-0.1)]	0.01
	2016	-0.1	[-0.3-0.1]	0.23	0.0	[-0.2-0.1]	0.65	0.0	[-0.2-0.1]	0.67
Age (<50 years)	50-59	0.5	[0.2-0.9]	<0.01	0.6	[0.3-1.0]	<0.01	0.6	[0.3-0.9]	<0.01
	60-69	0.8	[0.5-1.1]	<0.01	1.1	[0.7-1.4]	<0.01	1.0	[0.7-1.3]	<0.01
	70-79	<0.1	[-0.4-0.3]	0.82	0.4	[0.1-0.8]	0.01	0.4	[0.1-0.7]	0.02
	80-84	-1.0	[-1.3-(-0.6)]	<0.01	-0.4	[-0.7-0.0]	0.05	-0.4	[-0.8-<0.1]	0.03
	≥85	-1.6	[-2.0-(-1.2)]	<0.01	-1.0	[-1.4-(-0.5)]	<0.01	-1.0	[-1.4-(-0.6)]	<0.01
Sex (woman)	man	0.5	[0.1-0.8]	0.01	0.4	[<0.1-0.8]	0.03	0.4	[<0.1-0.7]	0.04
BMI, (kg/m^2)		-0.1	[-0.1-(-0.1)]	<0.01	-0.1	[-0.1-(-0.1)]	<0.01	-0.1	[-0.1-(-0.1)]	<0.01
Pre-operative ASA physical function score (Mild disease not incapacitating, grade 2)	Fit and healthy (grade 1)	1.0	[0.8-1.2]	<0.01	1.0	[0.8-1.1]	<0.01	0.9	[0.8-1.1]	<0.01

© 2019 Garriga C et al. *JAMA Network Open.*

	Incapacitating systemic disease (grade 3)	-1.6	[-1.8-(-1.4)]	<0.01	-1.5	[-1.7-(-1.4)]	<0.01	-1.4	[-1.6-(-1.3)]	<0.01
	Life threatening disease or Expected to die within 24h (grades 4 and 5)	-2.6	[-3.6-(-1.5)]	<0.01	-2.5	[-3.6-(-1.5)]	<0.01	-2.4	[-3.4-(-1.3)]	<0.01
IMD (Most deprived 20%)	Least deprived 20%	1.6	[1.4-1.8]	<0.01	1.5	[1.3-1.7]	<0.01	1.5	[1.3-1.7]	<0.01
	Less deprived 20-40%	1.3	[1.1-1.5]	<0.01	1.2	[1.0-1.4]	<0.01	1.2	[1.0-1.4]	<0.01
	Less deprived 40-60%	0.6	[0.4-0.8]	<0.01	0.6	[0.4-0.8]	<0.01	0.6	[0.4-0.8]	<0.01
	More deprived 20-40%	0.2	[<0.1-0.4]	0.03	0.2	[<0.1-0.5]	0.03	0.2	[<0.1-0.5]	0.02
Rural/Urban indicator (Urban >=10,000 habitants)	Town and fringe	0.4	[0.2-0.6]	<0.01	0.4	[0.2-0.6]	<0.01	0.4	[0.2-0.6]	<0.01

© 2019 Garriga C et al. *JAMA Network Open.*

	Village/isolated	0.8	[0.6-0.9]	<0.01	0.8	[0.6-0.9]	<0.01	0.7	[0.5-0.9]	<0.01
Primary indication (Osteoarthritis)	Osteoarthritis and Other*									
Charlson score (None, 0)	Mild (1)	-1.0	[-1.1-(-0.8)]	<0.01	-1.0	[-1.1-(-0.8)]	<0.01	-0.9	[-1.1-(-0.8)]	<0.01
	Moderate (2)	-1.2	[-1.4-(-0.9)]	<0.01	-1.1	[-1.4-(-0.9)]	<0.01	-1.1	[-1.3-(-0.8)]	<0.01
	Severe (≥3)	-1.1	[-1.5-(-0.8)]	<0.01	-1.1	[-1.4-(-0.8)]	<0.01	-1.0	[-1.4-(-0.7)]	<0.01
OHS baseline (points)		-0.8	[-0.8-(-0.8)]	<0.01	-0.8	[-0.8-(-0.8)]	<0.01	-0.8	[-0.8-(-0.8)]	<0.01
EQ-5D-3L (Highest quintile)	Lowest quintile	2.3	[2.0-2.5]	<0.01	2.3	[2.0-2.5]	<0.01	2.3	[2.0-2.5]	<0.01
	2nd quintile	3.3	[3.1-3.6]	<0.01	3.3	[3.0-3.6]	<0.01	3.3	[3.0-3.5]	<0.01
	3rd quintile	3.2	[2.9-3.5]	<0.01	3.2	[2.9-3.5]	<0.01	3.2	[2.9-3.4]	<0.01
	4th quintile	3.9	[3.6-4.2]	<0.01	3.9	[3.6-4.2]	<0.01	3.9	[3.6-4.2]	<0.01
Woman x EQ-5D-3L (Highest quintile)	Woman x Lowest quintile	0.9	[0.5-1.3]	<0.01	0.9	[0.5-1.3]	<0.01	0.9	[0.5-1.4]	<0.01
	Woman x 2nd quintile	0.3	[-0.1-0.7]	0.17	0.3	[-0.1-0.7]	0.16	0.3	[-0.1-0.8]	0.13
	Woman x 3rd quintile	0.7	[0.3-1.1]	<0.01	0.7	[0.3-1.1]	<0.01	0.7	[0.3-1.2]	<0.01

© 2019 Garriga C et al. *JAMA Network Open.*

	Woman x 4th quintile	0.5	[0.1-0.9]	0.03	0.5	[0.1-0.9]	0.03	0.5	[0.1-0.9]	0.02
Surgical factors										
Lead surgeon experience (Consultant)	Other**				-0.6	[-0.7-(-0.4)]	<0.01	-0.3	[-0.4-(-0.1)]	<0.01
Surgical volume per lead surgeon (>150 surgeries per year)	≤10				-1.2	[-1.6-(-0.8)]	<0.01	-1.0	[-1.5-(-0.6)]	<0.01
	11-50				-0.7	[-0.9-(-0.5)]	<0.01	-0.6	[-0.8-(-0.4)]	<0.01
	51-75				-0.5	[-0.7-(-0.4)]	<0.01	-0.5	[-0.7-(-0.3)]	<0.01
	76-100				-0.5	[-0.7-(-0.3)]	<0.01	-0.4	[-0.6-(-0.2)]	<0.01
	101-150				-0.3	[-0.5-(-0.1)]	<0.01	-0.3	[-0.5-(-0.1)]	<0.01
Surgical volume per unit (Surgeries per year, ≥500)	≤200				-0.2	[-0.4-<0.1]	0.13			
	200-299				-0.2	[-0.4-<0.1]	0.04			
	300-399				-0.1	[-0.3-0.1]	0.16			
	400-499				0.1	[-0.2-0.4]	0.53			

© 2019 Garriga C et al. *JAMA Network Open.*

Minimally invasive surgery (No)	Yes									
Thromboprophylaxis (LMWH +/-Other)	None									
	Aspirin only									
	Other (no LMWH)									
Mechanical prophylaxis (any)	None									
General anaesthesia (No)	Yes				-0.3	[-0.4-(-0.1)]	<0.01	-0.3	[-0.5-(-0.1)]	<0.01
Regional – epidural anaesthesia (No)	Yes									
Regional – nerve block anaesthesia (No)	Yes									
Regional – spinal anaesthesia (No)	Yes				0.4	[0.2-0.5]	<0.01	0.3	[0.2-0.5]	<0.01
Type of approach (Posterior)	Anterior, antero-lateral,				-0.9	[-1.0-(-0.8)]	<0.01	-0.9	[-1.0-(-0.7)]	<0.01

© 2019 Garriga C et al. *JAMA Network Open.*

	hardinge, lateral, trochanteric osteotomy, other									
Femoral bone graft (No)	Yes				-0.8	[-1.6-(-0.1)]	0.03	-0.8	[-1.5-<0.1)]	0.05
Cup bone graft (No)	Yes				0.5	[0.2-0.8]	<0.01	0.5	[0.2-0.8]	<0.01
Primary cup fixation (Cementless)	Cemented				-0.6	[-0.8-(-0.5)]	<0.01	-0.6	[-0.7-(-0.4)]	<0.01
Type of primary stem fixation (Cemented THR stem)	Not available or Resurfacing				<0.1	[-0.8-0.9]	0.91			
	Cementless THR stem				<0.1	[-0.1-0.1]	0.94			
Bearing surface (MoP)	MoM				-1.7	[-3.9-0.4]	0.11	-1.6	[-3.6-0.3]	0.10
	CoC				0.2	[<0.1-0.5]	0.03	0.3	[0.1-0.6]	<0.01
	CoP				0.2	[0.1-0.4]	<0.01	0.3	[0.1-0.4]	<0.01

© 2019 Garriga C et al. *JAMA Network Open.*

	CoM or MoC or unknown				-0.5	[-1.1-0.1]	0.07	-0.4	[-1.0-0.2]	0.20
Femoral head size (≤28mm)	32				0.1	[-0.1-0.2]	0.45	0.1	[-0.1-0.2]	0.32
	36-42				<0.1	[-0.2-0.2]	0.91	<0.1	[-0.2-0.2]	0.64
	≥44				2.0	[0.2-3.8]	0.03	2.1	[0.3-3.9]	0.02
Hospital organisation factors										
Unit type (Public hospital)	Private hospital							0.8	[0.6-0.9]	<0.01
	Independent sector (private) – treatment centre							1.0	[0.7-1.3]	<0.01
FTE of speciality groups on trauma and orthopaedic surgery (0-24 FTEs)	25-29									
	30-39									

© 2019 Garriga C et al. *JAMA Network Open.*

	40-49									
	>50									
Consultant FTE (0-24 FTEs)	25-29									
	30-39									
	40-49									
	>50									
middle grade doctor FTE (0-24 FTEs)	25-29									
	30-39									
	40-49									
	>50									
early career doctor FTE (0-24 FTEs)	25-29									
	30-39									
Total beds available overnight (0-349)	350-499							-0.5	[-0.8-(-0.2)]	<0.01
	500-699							-0.7	[-1.0-(-0.4)]	<0.01
	700-999							-0.7	[-1.0-(-0.4)]	<0.01

© 2019 Garriga C et al. *JAMA Network Open.*

	≥1000							-0.8	[-1.1-(-0.4)]	<0.01
Total beds available overnight for trauma and orthopaedic surgery (0-34)	35-49									
	50-69									
	70-99									
	≥100									
Total beds available overnight for rehabilitation (0)	>0-10									
	11-20									
	≥20									
Operating theatres (<10)	10-14							0.4	[0.1-0.7]	0.02
	15-19							0.7	[0.3-1.0]	<0.01
	20-24							0.6	[0.3-1.0]	<0.01
	≥25							0.6	[0.2-1.0]	<0.01

© 2019 Garriga C et al. *JAMA Network Open.*

Dedicated day case operating theatres (≥7)	0							-0.2	[-0.4-<0.1]	0.12
	1-2							-0.3	[-0.5-<0.1]	0.02
	3-4							<0.1	[-0.2-0.2]	0.89
	5-6							-0.1	[-0.3-0.1]	0.19

American Society of Anesthesiologists, ASA; Index of multiple deprivation, IMD; European Quality of life-five domain, EQ-5D-3L; low-molecular-weight heparin, LMWH; total hip replacement, THR; metal-on-metal, MoM; metal-on-polyethylene, MoP; ceramic-on-ceramic, CoC; ceramic-on-polyethylene, CoP; ceramic-on-metal, CoM; metal-on-ceramic, MoC; full time equivalent, FTE (proportion of full time contracted hours).

© 2019 Garriga C et al. *JAMA Network Open.*

eTable 8. Continuous Multilevel Models of Oxford Knee Score for Patients Who Underwent Planned Primary TKR/UKR

		Patient model			Surgical model			Hospital organisation model		
	Categories	Coef.	95% CI	P	Coef.	95% CI	P	Coef.	95% CI	P
Intercept		25.62	[24.89-26.35]	<0.01	25.96	[25.18-26.73]	<0.01	25.61	[24.84-26.38]	<0.01
Patient factors										
Calendar year (2016)	2015	-0.74	[-0.90-(-0.58)]	<0.01	-0.71	[-0.87-(-0.55)]	<0.01	-0.69	[-0.85-(-0.53)]	<0.01
	2016	-0.56	[-0.72-(-0.40)]	<0.01	-0.57	[-0.73-(-0.41)]	<0.01	-0.56	[-0.72-(-0.40)]	<0.01
Age (<50 years)	50-59	1.30	[0.76-1.85]	<0.01	1.30	[0.76-1.85]	<0.01	1.26	[0.71-1.81]	<0.01
	60-69	2.57	[2.04-3.10]	<0.01	2.56	[2.03-3.09]	<0.01	2.51	[1.98-3.03]	<0.01
	70-79	2.92	[2.39-3.45]	<0.01	2.92	[2.39-3.45]	<0.01	2.86	[2.33-3.39]	<0.01
	80-84	2.54	[1.99-3.09]	<0.01	2.53	[1.98-3.08]	<0.01	2.48	[1.93-3.03]	<0.01
	≥85	2.23	[1.64-2.82]	<0.01	2.21	[1.62-2.80]	<0.01	2.18	[1.59-2.77]	<0.01
Sex (woman)	man	0.38	[0.27-0.50]	<0.01	0.38	[0.26-0.49]	<0.01	0.38	[0.27-0.50]	<0.01
BMI, (kg/m^2)		-0.08	[-0.09-(-0.07)]	<0.01	-0.08	[-0.09-(-0.07)]	<0.01	-0.07	[-0.08-(-0.06)]	<0.01
Pre-operative ASA physical function score (Mild disease not incapacitating, grade 2)	Fit and healthy (grade 1)	0.80	[0.60-1.01]	<0.01	0.81	[0.60-1.01]	<0.01	0.77	[0.57-0.98]	<0.01

© 2019 Garriga C et al. *JAMA Network Open.*

	Incapacitating systemic disease (grade 3)	-1.16	[-1.32-(-1.00)]	<0.01	-1.13	[-1.29-(-0.97)]	<0.01	-1.05	[-1.21-(-0.90)]	<0.01
	Life threatening disease or Expected to die within 24h (grades 4 and 5)	-3.11	[-4.36-(-1.86)]	<0.01	-3.07	[-4.32-(-1.82)]	<0.01	-2.94	[-4.19-(-1.69)]	<0.01
IMD (Most deprived 20%)	Least deprived 20%	1.31	[1.10-1.52]	<0.01	1.30	[1.09-1.50]	<0.01	1.28	[1.07-1.49]	<0.01
	Less deprived 20-40%	1.08	[0.89-1.28]	<0.01	1.07	[0.87-1.26]	<0.01	1.05	[0.85-1.25]	<0.01
	Less deprived 40-60%	0.45	[0.25-0.65]	<0.01	0.44	[0.23-0.64]	<0.01	0.44	[0.23-0.64]	<0.01
	More deprived 20-40%	0.27	[0.07-0.48]	0.01	0.26	[0.06-0.47]	0.01	0.27	[0.06-0.47]	0.01
Rural/Urban indicator (Urban >=10,000 habitants)	Town and fringe	0.47	[0.28-0.67]	<0.01	0.48	[0.29-0.68]	<0.01	0.48	[0.28-0.67]	<0.01

© 2019 Garriga C et al. *JAMA Network Open.*

	Village/isolated	0.84	[0.65-1.03]	<0.01	0.85	[0.66-1.04]	<0.01	0.83	[0.64-1.02]	<0.01
Primary indication (Osteoarthritis)	Osteoarthritis and Other*									
Charlson score (None, 0)	Mild (1)	-1.01	[-1.15-(-0.88)]	<0.01	-1.02	[-1.15-(-0.88)]	<0.01	-1.00	[-1.13-(-0.86)]	<0.01
	Moderate (2)	-0.97	[-1.19-(-0.75)]	<0.01	-0.96	[-1.18-(-0.74)]	<0.01	-0.91	[-1.13-(-0.69)]	<0.01
	Severe (≥3)	-1.28	[-1.59-(-0.97)]	<0.01	-1.27	[-1.58-(-0.96)]	<0.01	-1.22	[-1.53-(-0.91)]	<0.01
OKS baseline (points)		-0.67	[-0.68-(-0.66)]	<0.01	-0.68	[-0.69-(-0.67)]	<0.01	-0.68	[-0.69-(-0.67)]	<0.01
EQ-5D-3L (Lowest quintile)	2nd quintile	2.68	[2.48-2.88]	<0.01	2.67	[2.47-2.87]	<0.01	2.66	[2.46-2.86]	<0.01
	3rd quintile	3.06	[2.88-3.25]	<0.01	3.04	[2.86-3.23]	<0.01	3.02	[2.84-3.21]	<0.01
	4th quintile	2.43	[2.19-2.68]	<0.01	2.42	[2.17-2.66]	<0.01	2.40	[2.15-2.64]	<0.01
	Highest quintile	3.81	[3.60-4.01]	<0.01	3.78	[3.57-3.99]	<0.01	3.77	[3.56-3.98]	<0.01
Surgical factors										
Lead surgeon experience (Consultant)	Other**				-0.49	[-0.63-(-0.34)]	<0.01	-0.28	[-0.43-(-0.12)]	<0.01

© 2019 Garriga C et al. *JAMA Network Open.*

Surgical volume per lead surgeon (>150 surgeries per year)	≤10				-0.54	[-1.01-(-0.06)]	0.03			
	11-50				-0.33	[-0.52-(-0.13)]	<0.01			
	51-75				-0.23	[-0.43-(-0.03)]	0.03			
	76-100				-0.08	[-0.27-0.12]	0.43			
	101-150				-0.16	[-0.35-0.03]	0.10			
Surgical volume per unit (Surgeries per year, ≥500)	≤200				-0.19	[-0.42-0.03]	0.10	-0.43	[-0.66-(-0.20)]	<0.01
	200-299				-0.20	[-0.40-0.01]	0.06	-0.29	[-0.50-(-0.08)]	0.01
	300-399				-0.04	[-0.23-0.16]	0.71	-0.08	[-0.28-0.11]	0.40
	400-499				-0.37	[-0.59-(-0.15)]	<0.01	-0.40	[-0.62-(-0.19)]	<0.01
Minimally invasive surgery (No)	Yes									
Thromboprophylaxis (LMWH +/-Other)	None									
	Aspirin only									

© 2019 Garriga C et al. *JAMA Network Open.*

	Other (no LMWH)									
Mechanical prophylaxis (any)	None				-0.37	[-0.71-(-0.03)]	0.04			
General anaesthesia (No)	Yes				-0.41	[-0.59-(-0.23)]	<0.01	-0.46	[-0.64-(-0.28)]	<0.01
Regional – epidural anaesthesia (No)	Yes									
Regional – nerve block anaesthesia (No)	Yes									
Regional – spinal anaesthesia (No)	Yes				0.29	[0.10-0.48]	<0.01	0.25	[0.07-0.44]	0.01
Type of approach (Medial parapatellar)	Lateral parapatellar				0.40	[-0.23-1.02]	0.21	0.45	[-0.17-1.08]	0.16
	Mid-Vastus				0.17	[-0.18-0.52]	0.34	0.20	[-0.15-0.56]	0.26
	Sub-Vastus				0.67	[0.11-1.24]	0.02	0.63	[0.07-1.19]	0.03
	Other				-0.10	[-0.59-0.39]	0.70	-0.10	[-0.59-0.39]	0.70
Femoral bone graft (No)	Yes									

© 2019 Garriga C et al. *JAMA Network Open.*

Tibia bone graft (No)	Yes									
Primary femoral fixation (Cemented)	Cementless									
Primary Tibial fixation (Cemented)	Cementless									
Type of knee implant (TKR)	UKR									
Hospital organisation factors										
Unit type (Public hospital)	Private hospital							0.73	[0.57-0.88]	<0.01
	Independent sector (private) – treatment centre							0.57	[0.28-0.85]	<0.01
FTE of speciality groups on trauma and orthopaedic surgery (0-24 FTEs)	25-29									

© 2019 Garriga C et al. *JAMA Network Open.*

	30-39									
	40-49									
	>50									
Consultant FTE (0-24 FTEs)	25-29									
	30-39									
	40-49									
	>50									
middle grade doctor FTE (0-24 FTEs)	25-29									
	30-39									
	40-49									
	>50									
early career doctor FTE (0-24 FTEs)	25-29									
	30-39									
Total beds available overnight (0-349)	350-499									
	500-699									

© 2019 Garriga C et al. *JAMA Network Open.*

	700-999									
	≥1000									
Total beds available overnight for trauma and orthopaedic surgery (0-34)	35-49									
	50-69									
	70-99									
	≥100									
Total beds available overnight for rehabilitation (0)	>0-10									
	11-20									
	≥20									
Operating theatres (<10)	10-14									
	15-19									
	20-24									
	≥25									

© 2019 Garriga C et al. *JAMA Network Open.*

Dedicated day case operating theatres (≥7)	0									
	1-2									
	3-4									
	5-6									

Total knee replacement, TKR; Unicompartmental knee replacement, UKR; American Society of Anesthesiologists, ASA; Index of multiple deprivation, IMD; European Quality of life-five domain, EQ-5D-3L; low-molecular-weight heparin, LMWH; full time equivalent, FTE (proportion of full time contracted hours).

© 2019 Garriga C et al. *JAMA Network Open.*

eTable 9. Logistic Multilevel Models of Complications at 6 Months for Patients Who Underwent Planned Primary Hip Replacement

		**Patient model**			**Surgical model**			**Hospital organisation model**		
	Categories	Coef.	95% CI	P	Coef.	95% CI	P	Coef.	95% CI	P
Intercept		-4.95	[-5.24-(-4.65)]	<0.01	-4.85	[-5.16-(-4.54)]	<0.01	-4.84	[-5.16-(-4.53)]	<0.01
Patient factors										
Calendar year (2016)	2015	0.10	[0.04-0.16]	<0.01	0.08	[0.02-0.14]	0.01	0.08	[0.02-0.14]	0.01
	2016	0.11	[0.05-0.17]	<0.01	0.11	[0.05-0.17]	<0.01	0.11	[0.05-0.17]	<0.01
Age (<50 years)	50-59	0.08	[-0.10-0.25]	0.38	0.07	[-0.10-0.25]	0.41	0.08	[-0.10-0.25]	0.40
	60-69	0.22	[0.06-0.39]	0.01	0.20	[0.04-0.37]	0.02	0.21	[0.04-0.37]	0.02
	70-79	0.55	[0.39-0.71]	<0.01	0.50	[0.33-0.67]	<0.01	0.50	[0.33-0.67]	<0.01
	80-84	0.86	[0.69-1.03]	<0.01	0.81	[0.63-0.99]	<0.01	0.81	[0.63-0.99]	<0.01
	≥85	1.16	[0.98-1.33]	<0.01	1.11	[0.92-1.29]	<0.01	1.10	[0.91-1.28]	<0.01
Sex (woman)	man	0.25	[0.20-0.30]	<0.01	0.27	[0.21-0.32]	<0.01	0.27	[0.22-0.32]	<0.01
BMI, (kg/m^2)		0.01	[0.01-0.02]	<0.01	0.01	[0.01-0.02]	<0.01	0.01	[0.01-0.02]	<0.01
Pre-operative ASA physical function score (Mild disease not incapacitating, grade 2)	Fit and healthy (grade 1)	-0.22	[-0.32-(-0.12)]	<0.01	-0.21	[-0.31-(-0.11)]	<0.01	-0.21	[-0.31-(-0.10)]	<0.01

© 2019 Garriga C et al. *JAMA Network Open.*

	Incapacitating systemic disease (grade 3)	0.43	[0.37-0.49]	<0.01	0.42	[0.37-0.48]	<0.01	0.41	[0.35-0.47]	<0.01
	Life threatening disease or Expected to die within 24h (grades 4 and 5)	0.85	[0.63-1.08]	<0.01	0.83	[0.61-1.06]	<0.01	0.82	[0.59-1.04]	<0.01
IMD (Most deprived 20%)	Least deprived 20%	-0.04	[-0.12-0.05]	0.38	-0.02	[-0.11-0.06]	0.56	-0.02	[-0.11-0.06]	0.60
	Less deprived 20-40%	-0.05	[-0.13-0.03]	0.21	-0.04	[-0.13-0.04]	0.30	-0.04	[-0.12-0.04]	0.34
	Less deprived 40-60%	0.08	[-0.01-0.16]	0.07	0.08	[<0.01-0.16]	0.06	0.08	[<0.01-0.17]	0.06
	More deprived 20-40%	0.09	[0.01-0.18]	0.03	0.10	[0.01-0.18]	0.03	0.10	[0.01-0.18]	0.03
Rural/Urban indicator (Urban >=10,000 habitants)	Town and fringe									

© 2019 Garriga C et al. *JAMA Network Open.*

	Village/isolated									
Primary indication (Osteoarthritis)	Osteoarthritis and Other*	0.36	[0.23-0.49]	<0.01	0.35	[0.23-0.48]	<0.01	0.35	[0.22-0.47]	<0.01
Charlson score (None, 0)	Mild (1)	0.33	[0.27-0.39]	<0.01	0.33	[0.27-0.39]	<0.01	0.33	[0.27-0.39]	<0.01
	Moderate (2)	0.50	[0.41-0.58]	<0.01	0.50	[0.41-0.58]	<0.01	0.49	[0.41-0.57]	<0.01
	Severe (≥3)	0.75	[0.66-0.85]	<0.01	0.75	[0.65-0.84]	<0.01	0.74	[0.64-0.83]	<0.01
EQ-5D-3L (Highest quintile)	Lowest quintile	-0.14	[-0.21-(-0.06)]	<0.01	-0.14	[-0.21-(-0.06)]	<0.01	-0.14	[-0.21-(-0.06)]	<0.01
	2nd quintile	-0.28	[-0.36-(-0.20)]	<0.01	-0.28	[-0.36-(-0.19)]	<0.01	-0.27	[-0.36-(-0.19)]	<0.01
	3rd quintile	-0.29	[-0.37-(-0.20)]	<0.01	-0.28	[-0.37-(-0.19)]	<0.01	-0.28	[-0.36-(-0.19)]	<0.01
	4th quintile	-0.36	[-0.45-(-0.27)]	<0.01	-0.35	[-0.44-(-0.26)]	<0.01	-0.35	[-0.43-(-0.26)]	<0.01
Surgical factors										
Lead surgeon experience (Consultant)	Other**									
Surgical volume per lead surgeon (>150 surgeries per year)	≤10									

© 2019 Garriga C et al. *JAMA Network Open.*

	11-50									
	51-75									
	76-100									
	101-150									
Surgical volume per unit (Surgeries per year, ≥500)	≤200				0.12	[0.03-0.20]	0.01	0.12	[0.04-0.21]	0.01
	200-299				0.08	[<0.01-0.17]	0.06	0.08	[-0.01-0.17]	0.07
	300-399				0.07	[-0.01-0.16]	0.10	0.08	[-0.01-0.17]	0.09
	400-499				0.05	[-0.09-0.18]	0.48	0.05	[-0.09-0.18]	0.48
Minimally invasive surgery (No)	Yes				-0.28	[-0.43-(-0.13)]	<0.01	-0.27	[-0.43-(-0.12)]	<0.01
Thromboprophylaxis (LMWH +/-Other)	None				0.14	[-0.12-0.40]	0.29	0.15	[-0.10-0.41]	0.25
	Aspirin only				0.19	[0.04-0.34]	0.01	0.19	[0.04-0.34]	0.01
	Other (no LMWH)				-0.04	[-0.10-0.02]	0.15	-0.03	[-0.09-0.03]	0.28
Mechanical prophylaxis (any)	None									

© 2019 Garriga C et al. *JAMA Network Open.*

General anaesthesia (No)	Yes									
Regional – epidural anaesthesia (No)	Yes									
Regional – nerve block anaesthesia (No)	Yes									
Regional – spinal anaesthesia (No)	Yes				-0.08	[-0.13-(-0.02)]	0.01	-0.07	[-0.13-(-0.01)]	0.02
Type of approach (Posterior)	Anterior, antero-lateral, hardinge, lateral, trochanteric osteotomy, other									
Femoral bone graft (No)	Yes									
Cup bone graft (No)	Yes									

© 2019 Garriga C et al. *JAMA Network Open.*

Primary cup fixation (Cementless)	Cemented									
Type of primary stem fixation (Cemented THR stem)	Not available or Resurfacing									
	Cementless THR stem									
Bearing surface (MoP)	MoM				-0.20	[-1.17-0.77]	0.69	-0.21	[-1.17-0.76]	0.68
	CoC				-0.04	[-0.15-0.07]	0.44	-0.05	[-0.16-0.06]	0.38
	CoP				-0.08	[-0.15-(-0.01)]	0.03	-0.08	[-0.16-(-0.01)]	0.02
	CoM or MoC or unknown				0.13	[-0.09-0.36]	0.25	0.12	[-0.10-0.35]	0.28
Femoral head size (≤28mm)	32				-0.10	[-0.16-(-0.04)]	<0.01	-0.10	[-0.16-(-0.04)]	<0.01
	36-42				-0.06	[-0.13-0.02]	0.17	-0.06	[-0.14-0.02]	0.14
	≥44				-0.13	[-0.98-0.73]	0.77	-0.13	[-0.99-0.73]	0.77
Hospital organisation factors										

© 2019 Garriga C et al. *JAMA Network Open.*

Unit type (Public hospital)	Private hospital							-0.08	[-0.15-(-0.01)]	0.03
	Independent sector (private) – treatment centre							-0.13	[-0.27-0.01]	0.06
FTE of speciality groups on trauma and orthopaedic surgery (0-24 FTEs)	25-29									
	30-39									
	40-49									
	>50									
Consultant FTE (0-24 FTEs)	25-29									
	30-39									
	40-49									
	>50									

© 2019 Garriga C et al. *JAMA Network Open.*

middle grade doctor FTE (0-24 FTEs)	25-29							0.09	[<0.01-0.17]	0.04
	30-39							0.05	[-0.03-0.13]	0.19
	40-49							0.07	[-0.03-0.16]	0.17
	>50							0.03	[-0.05-0.12]	0.45
early career doctor FTE (0-24 FTEs)	25-29									
	30-39									
Total beds available overnight (0-349)	350-499									
	500-699									
	700-999									
	≥1000									
Total beds available overnight for trauma and orthopaedic surgery (0-34)	35-49									
	50-69									
	70-99									

© 2019 Garriga C et al. *JAMA Network Open.*

	≥100									
Total beds available overnight for rehabilitation (0)	>0-10									
	11-20									
	≥20									
Operating theatres (<10)	10-14									
	15-19									
	20-24									
	≥25									
Dedicated day case operating theatres (≥7)	0									
	1-2									
	3-4									
	5-6									

American Society of Anesthesiologists, ASA; Index of multiple deprivation, IMD; European Quality of life-five domain, EQ-5D-3L; low-molecular-weight heparin, LMWH; total hip replacement, THR; metal-on-metal, MoM; metal-on-polyethylene, MoP; ceramic-on-ceramic, CoC; ceramic-on-polyethylene, CoP; ceramic-on-metal, CoM; metal-on-ceramic, MoC; full time equivalent, FTE (proportion of full time contracted hours).

© 2019 Garriga C et al. *JAMA Network Open.*

eTable 10. Logistic Multilevel Models of Complications at 6 Months for Patients Who Underwent Planned Primary Hip TKR/UKR

		**Patient model**			**Surgical model**			**Hospital organisation model**		
	Categories	Coef.	95% CI	P	Coef.	95% CI	P	Coef.	95% CI	P
Intercept		-3.92	[-4.20-(-3.65)]	<0.01	-4.40	[-4.70-(-4.10)]	<0.01	-4.39	[-4.70-(-4.09)]	<0.01
Patient factors										
Calendar year (2016)	2015	0.15	[0.09-0.20]	<0.01	0.14	[0.09-0.20]	<0.01	0.14	[0.08-0.19]	<0.01
	2016	0.15	[0.09-0.20]	<0.01	0.14	[0.09-0.20]	<0.01	0.14	[0.08-0.20]	<0.01
Age (<50 years)	50-59	-0.18	[-0.35-<0.01]	0.05	-0.20	[-0.38-(-0.03)]	0.03	-0.20	[-0.38-(-0.02)]	0.03
	60-69	-0.21	[-0.38-(-0.04)]	0.01	-0.27	[-0.44-(-0.10)]	<0.01	-0.26	[-0.43-(-0.09)]	<0.01
	70-79	-0.01	[-0.18-0.16]	0.94	-0.07	[-0.24-0.10]	0.44	-0.06	[-0.23-0.11]	0.49
	80-84	0.25	[0.08-0.43]	0.01	0.19	[0.01-0.37]	0.04	0.19	[0.02-0.37]	0.03
	≥85	0.61	[0.43-0.80]	<0.01	0.55	[0.36-0.73]	<0.01	0.55	[0.37-0.73]	<0.01
Sex (woman)	man	0.23	[0.19-0.28]	<0.01	0.24	[0.20-0.29]	<0.01	0.24	[0.20-0.29]	<0.01
BMI, (kg/m^2)										
Pre-operative ASA physical function score (Mild disease not incapacitating, grade 2)	Fit and healthy (grade 1)	-0.22	[-0.32-(-0.12)]	<0.01	-0.20	[-0.30-(-0.10)]	<0.01	-0.19	[-0.29-(-0.09)]	<0.01

© 2019 Garriga C et al. *JAMA Network Open.*

	Incapacitating systemic disease (grade 3)	0.42	[0.36-0.47]	<0.01	0.41	[0.35-0.46]	<0.01	0.39	[0.34-0.45]	<0.01
	Life threatening disease or Expected to die within 24h (grades 4 and 5)	0.94	[0.68-1.20]	<0.01	0.90	[0.64-1.16]	<0.01	0.88	[0.62-1.14]	<0.01
IMD (Most deprived 20%)	Least deprived 20%	-0.13	[-0.20-(-0.05)]	<0.01	-0.11	[-0.19-(-0.03)]	0.01	-0.11	[-0.18-(-0.03)]	0.01
	Less deprived 20-40%	-0.08	[-0.15-(-0.01)]	0.03	-0.07	[-0.14-<0.01]	0.07	-0.06	[-0.14-0.01]	0.09
	Less deprived 40-60%	-0.03	[-0.10-0.04]	0.44	-0.03	[-0.10-0.05]	0.46	-0.03	[-0.10-0.05]	0.48
	More deprived 20-40%	0.03	[-0.04-0.10]	0.43	0.03	[-0.04-0.10]	0.40	0.03	[-0.04-0.10]	0.43
Rural/Urban indicator (Urban >=10,000 habitants)	Town and fringe									

© 2019 Garriga C et al. *JAMA Network Open.*

	Village/isolated									
Primary indication (Osteoarthritis)	Osteoarthritis and Other*	0.26	[0.07-0.44]	0.01	0.24	[0.05-0.43]	0.01	0.23	[0.04-0.41]	0.02
Charlson score (None, 0)	Mild (1)	0.28	[0.22-0.33]	<0.01	0.28	[0.23-0.33]	<0.01	0.27	[0.22-0.33]	<0.01
	Moderate (2)	0.41	[0.34-0.49]	<0.01	0.41	[0.34-0.49]	<0.01	0.40	[0.33-0.48]	<0.01
	Severe (≥3)	0.69	[0.60-0.78]	<0.01	0.69	[0.60-0.78]	<0.01	0.68	[0.58-0.77]	<0.01
EQ-5D-3L (Lowest quintile)	2nd quintile	-0.08	[-0.15-<0.01]	0.04	-0.07	[-0.15-<0.01]	0.05	-0.07	[-0.15-<0.01]	0.06
	3rd quintile	-0.18	[-0.25-(-0.12)]	<0.01	-0.18	[-0.24-(-0.11)]	<0.01	-0.17	[-0.24-(-0.11)]	<0.01
	4th quintile	-0.21	[-0.29-(-0.12)]	<0.01	-0.20	[-0.29-(-0.11)]	<0.01	-0.19	[-0.28-(-0.10)]	<0.01
	Highest quintile	-0.26	[-0.33-(-0.20)]	<0.01	-0.25	[-0.32-(-0.19)]	<0.01	-0.25	[-0.31-(-0.18)]	<0.01
Surgical factors										
Lead surgeon experience (Consultant)	Other**									
Surgical volume per lead surgeon (>150 surgeries per year)	≤10				-0.10	[-0.31-0.10]	0.32			

© 2019 Garriga C et al. *JAMA Network Open.*

	11-50				0.08	[<0.01-0.16]	0.06			
	51-75				0.07	[-0.01-0.15]	0.09			
	76-100				0.06	[-0.02-0.14]	0.15			
	101-150				0.08	[<0.01-0.16]	0.04			
Surgical volume per unit (Surgeries per year, ≥500)	≤200				0.09	[0.01-0.18]	0.03	0.09	[0.01-0.18]	0.03
	200-299				0.08	[0.01-0.16]	0.03	0.08	[<0.01-0.16]	0.04
	300-399				0.08	[<0.01-0.16]	0.05	0.08	[<0.01-0.16]	0.04
	400-499				0.05	[-0.03-0.14]	0.24	0.05	[-0.04-0.13]	0.27
Minimally invasive surgery (No)	Yes									
Thromboprophylaxis (LMWH +/-Other)	None				-0.27	[-0.53-(-0.01)]	0.05			
	Aspirin only				-0.04	[-0.19-0.10]	0.57			
	Other (no LMWH)				-0.03	[-0.09-0.03]	0.27			
Mechanical prophylaxis (any)	None				0.13	[<0.01-0.27]	0.05			

© 2019 Garriga C et al. *JAMA Network Open.*

General anaesthesia (No)	Yes				0.09	[0.04-0.14]	<0.01	0.08	[0.03-0.13]	<0.01
Regional – epidural anaesthesia (No)	Yes									
Regional – nerve block anaesthesia (No)	Yes									
Regional – spinal anaesthesia (No)	Yes									
Type of approach (Medial parapatellar)	Lateral parapatellar									
	Mid-Vastus									
	Sub-Vastus									
	Other									
Femoral bone graft (No)	Yes									
Tibia bone graft (No)	Yes									
Primary femoral fixation (Cemented)	Cementless									

© 2019 Garriga C et al. *JAMA Network Open.*

Primary Tibial fixation (Cemented)	Cementless									
Type of knee implant (TKR)	UKR				0.41	[0.30-0.52]	<0.01	0.41	[0.30-0.52]	<0.01
Hospital organisation factors										
Unit type (Public hospital)	Private hospital							-0.10	[-0.16-(-0.04)]	<0.01
	Independent sector (private) – treatment centre							-0.30	[-0.42-(-0.17)]	<0.01
FTE of speciality groups on trauma and orthopaedic surgery (0-24 FTEs)	25-29									
	30-39									
	40-49									
	>50									

© 2019 Garriga C et al. *JAMA Network Open.*

Consultant FTE (0-24 FTEs)	25-29									
	30-39									
	40-49									
	>50									
middle grade doctor FTE (0-24 FTEs)	25-29							-0.01	[-0.10-0.07]	0.80
	30-39							0.03	[-0.06-0.11]	0.54
	40-49							0.11	[-0.01-0.22]	0.07
	>50							0.10	[<0.01-0.21]	0.05
early career doctor FTE (0-24 FTEs)	25-29									
	30-39									
Total beds available overnight (0-349)	350-499									
	500-699									
	700-999									
	≥1000									

© 2019 Garriga C et al. *JAMA Network Open.*

Total beds available overnight for trauma and orthopaedic surgery (0-34)	35-49									
	50-69									
	70-99									
	≥100									
Total beds available overnight for rehabilitation (0)	>0-10									
	11-20									
	≥20									
Operating theatres (<10)	10-14									
	15-19									
	20-24									
	≥25									
Dedicated day case operating theatres (≥7)	0							0.12	[0.04-0.20]	<0.01

© 2019 Garriga C et al. *JAMA Network Open.*

	1-2							0.05	[-0.02-0.13]	0.18
	3-4							0.10	[0.02-0.17]	0.02
	5-6							0.11	[0.02-0.20]	0.01

Total knee replacement, TKR; Unicompartmental knee replacement, UKR; American Society of Anesthesiologists, ASA; Index of multiple deprivation, IMD; European Quality of life-five domain, EQ-5D-3L; low-molecular-weight heparin, LMWH; full time equivalent, FTE (proportion of full time contracted hours).

© 2019 Garriga C et al. *JAMA Network Open.*

eTable 11. Estimation of Patient Outcomes of Hip Replacement, TKR, and UKR, Adjusted for Patient, Surgical, and Hospital Organizational Factors

**Mean LOS (days)**							
Hip replacements				TKRs/UKRs			
5 shortest		*5 longest*		*5 shortest*		*5 longest*	
CCG	Predicted (SD)	CCG	Predicted (SD)	CCG	Predicted (SD)	CCG	Predicted (SD)
Scarborough and Ryedale	2.7 (1.0)	Hillingdon	6.1 (1.8)	Scarborough and Ryedale	2.9 (1.0)	West London	6.6 (1.7)
Hastings and Rother	2.9 (1.0)	Harrow	6.1 (2.1)	Northumberland	3.2 (0.7)	Hammersmith and Fulham	6.6 (1.8)
Hambleton, Richmondshire and Whitby	3.1 (0.8)	Camden	6.1 (1.9)	Bassetlaw	3.3 (1.4)	Camden	6.5 (1.8)
Northumberland	3.1 (0.9)	Barnet	5.8 (2.0)	Hastings and Rother	3.4 (1.0)	Central London (Westminster)	6.1 (1.6)
High Weald Lewes Havens	3.3 (1.4)	Central London (Westminster)	5.7 (2.0)	Salford	3.4 (1.0)	Barnet	6.1 (1.6)
**Mean of bed-day costs (£)**							
Hip replacements				TKRs/UKRs			

© 2019 Garriga C et al. *JAMA Network Open.*

5 lowest		*5 highest*		*5 lowest*		*5 highest*	
CCG	Predicted (SD)	CCG	Predicted (SD)	CCG	Predicted (SD)	CCG	Predicted (SD)
Scarborough and Ryedale	4727 (1026)	Hillingdon	8800 (1572)	Scarborough and Ryedale	4758 (1096)	Central London (Westminster)	8692 (1507)
Hastings and Rother	4880 (1051)	Harrow	8647 (1881)	Northumberland	4930 (720)	City and Hackney	8686 (1247)
Northumberland	4998 (867)	Camden	8250 (1561)	Bassetlaw	4960 (1329)	Lewisham	8676 (1585)
Hambleton, Richmondshire and Whitby	5187 (908)	Barnet	8132 (1788)	Hastings and Rother	5294 (1050)	West London	8639 (1438)
High Weald Lewes Havens	5219 (1305)	Waltham Forest	8060 (1710)	Salford	5344 (1008)	Camden	8600 (1493)
**Mean OHS change (points)**				**Mean OKS change (points)**			
5 highest		*5 lowest*		*5 highest*		*5 lowest*	
CCG	Predicted (SD)	CCG	Predicted (SD)	CCG	Predicted (SD)	CCG	Predicted (SD)

© 2019 Garriga C et al. *JAMA Network Open.*

Mid Essex	24.6 (5.3)	Brent	18.7 (6.2)	Ipswich and East Suffolk	18.8 (4.2)	City and Hackney	13.1 (4.3)
Ipswich and East Suffolk	24.1 (4.9)	Islington	19.1 (6.4)	Eastern Cheshire	18.5 (4.2)	Newham	13.4 (4.3)
Stoke on Trent	24.1 (5.0)	Harrow	19.2 (6.6)	Castle Point and Rochford	18.5 (4.0)	Islington	13.6 (5.4)
Scarborough and Ryedale	23.7 (5.4)	Kingston	19.4 (6.6)	Warrington	18.5 (4.2)	Brighton and Hove	13.7 (4.5)
Nene	23.6 (5.3)	Croydon	19.4 (5.9)	Nene	18.5 (4.2)	Barnet	13.8 (4.7)
**Mean of complications at 6 months (%)**							
Hip replacements				TKRs/UKRs			
5 lowest		*5 highest*		*5 lowest*		*5 highest*	
CCG	Predicted (SD)	CCG	Predicted (SD)	CCG	Predicted (SD)	CCG	Predicted (SD)
High Weald Lewes Havens	3.0 (2.4)	Tower Hamlets	5.4 (4.1)	Hull	2.9 (1.4)	Herts Valleys	5.8 (2.7)
Leeds West	3.1 (1.9)	Newham	5.3 (3.2)	Dartford, Gravesham and Swanley	2.9 (1.4)	South Devon and Torbay	5.8 (3.2)

© 2019 Garriga C et al. *JAMA Network Open.*

Solihull	3.2 (2.0)	Camden	5.2 (3.4)	Calderdale	2.9 (1.5)	Tower Hamlets	5.5 (2.4)
Herefordshire	3.2 (2.0)	Enfield	5.1 (3.0)	Erewash	3.1 (1.6)	Camden	5.4 (2.8)
West Leicestershire	3.2 (2.1)	Hillingdon	5.0 (3.3)	Hambleton, Richmondshire and Whitby	3.1 (1.4)	Newham	5.4 (2.7)

Total knee replacement, TKR; Unicompartmental knee replacement, UKR; standard deviation, SD; Oxford hip score, OHS; Oxford knee score, OKS.

© 2019 Garriga C et al. *JAMA Network Open.*

eFigure 1. Flow Diagram Showing Selection of Patients for Inclusion in This Study

Blue shows inclusion, and orange shows exclusion.

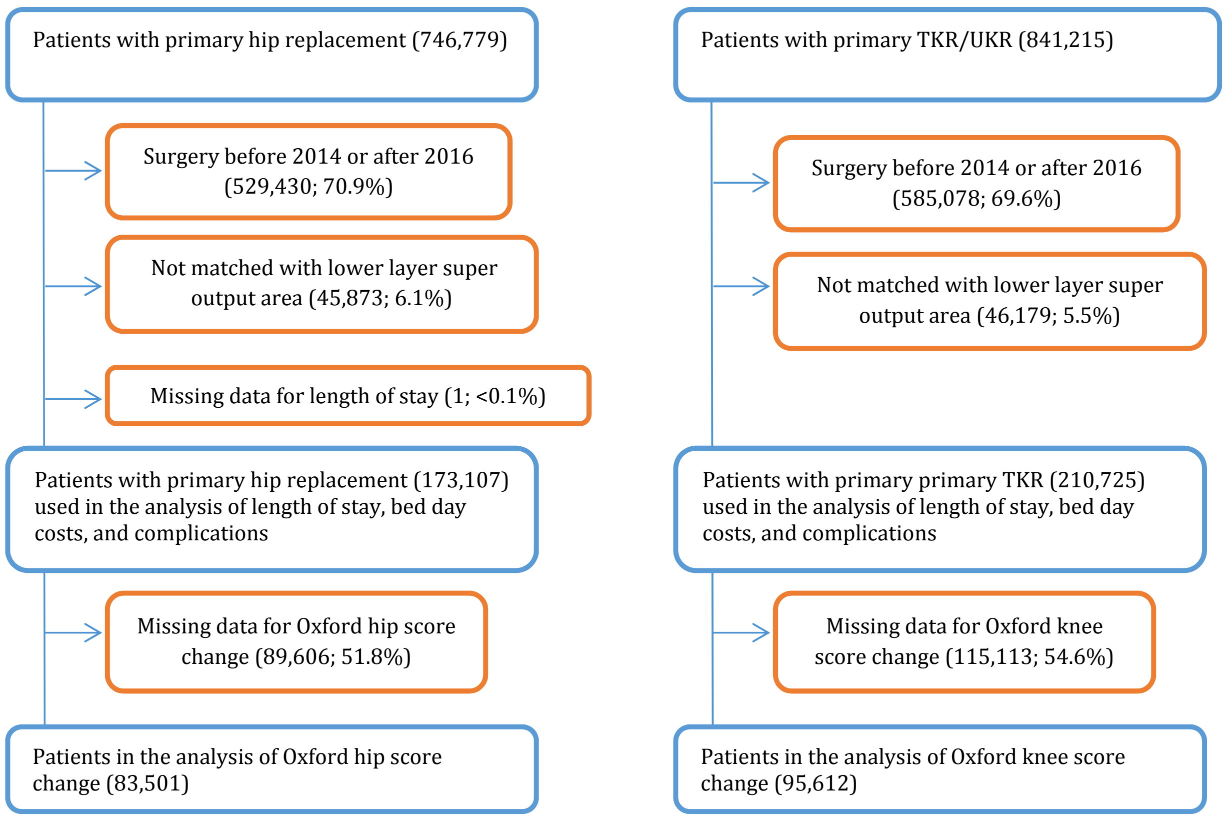

Total knee replacement, TKR; unicompartmental knee replacement, UKR.

© 2019 Garriga C et al. *JAMA Network Open.*

eFigure 2. Caterpillar Plots of Patient Outcomes for Primary Knee Replacement by Health Area in England, 2014-2016

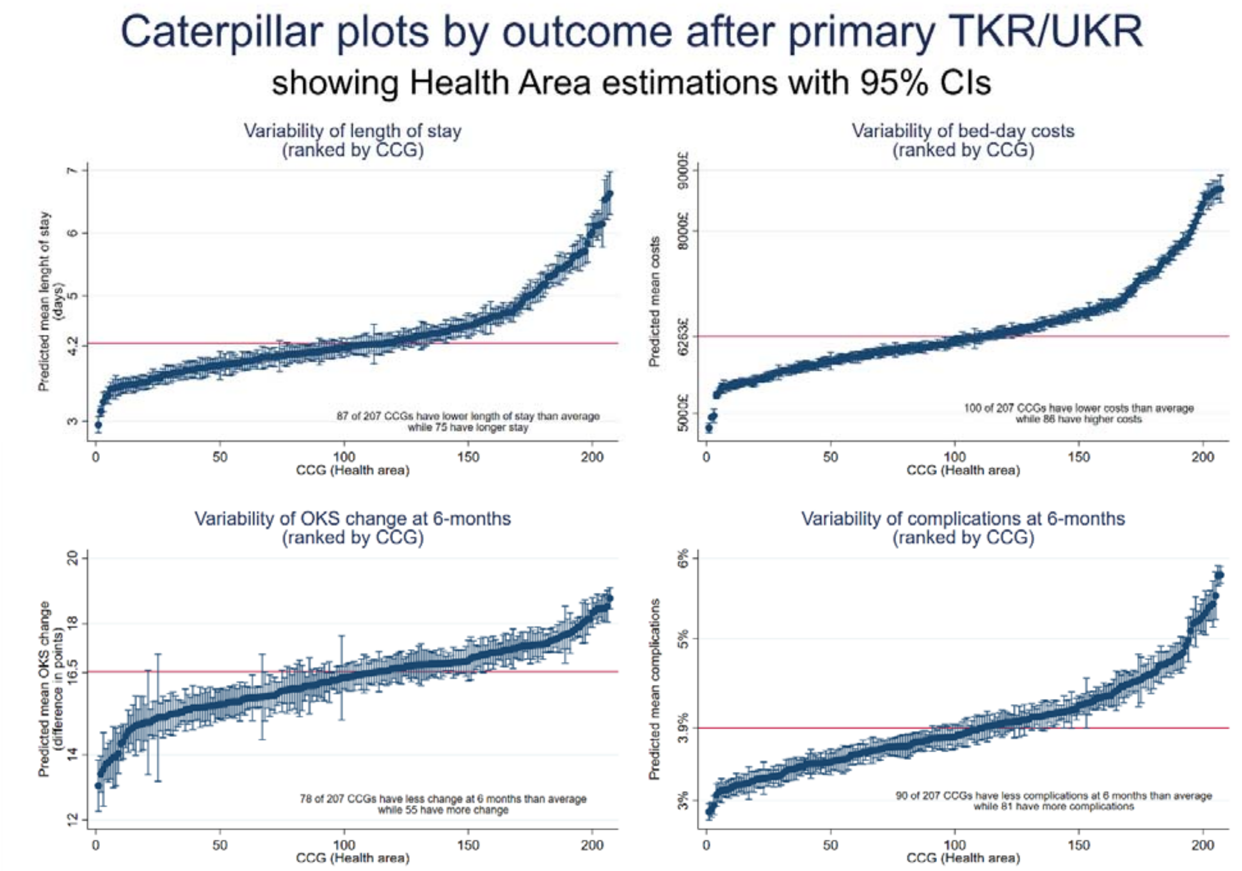

Horizontal red line is the predicted mean value for the sample. Confidence intervals, CI; clinical commissioning group, CCG; total knee replacement, TKR; unicompartmental knee replacement, UKR.; Oxford knee score, OKS.

© 2019 Garriga C et al. *JAMA Network Open.*

eFigure 3. Trends for 5 Worst and Best Health Areas (Clinical Commissioning Group Areas) for Primary Hip Replacement, England, 2014-2016

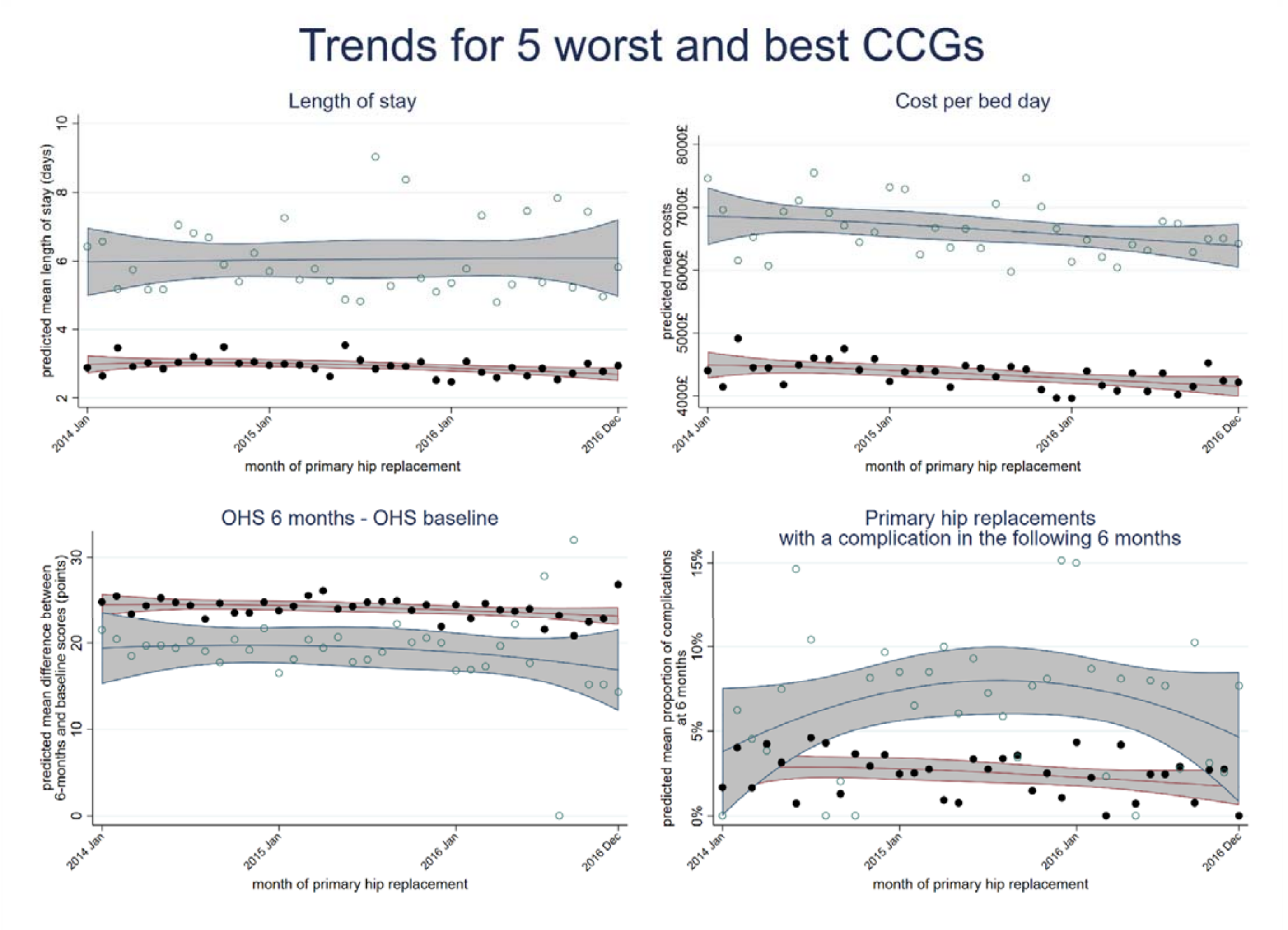

Oxford hip score, OHS; clinical commissioning group, CCG.

© 2019 Garriga C et al. *JAMA Network Open.*

eFigure 4. Trends for 5 Worst and Best Health Areas (Clinical Commissioning Group Areas) for Primary TKR/UKR, England, 2014-2016

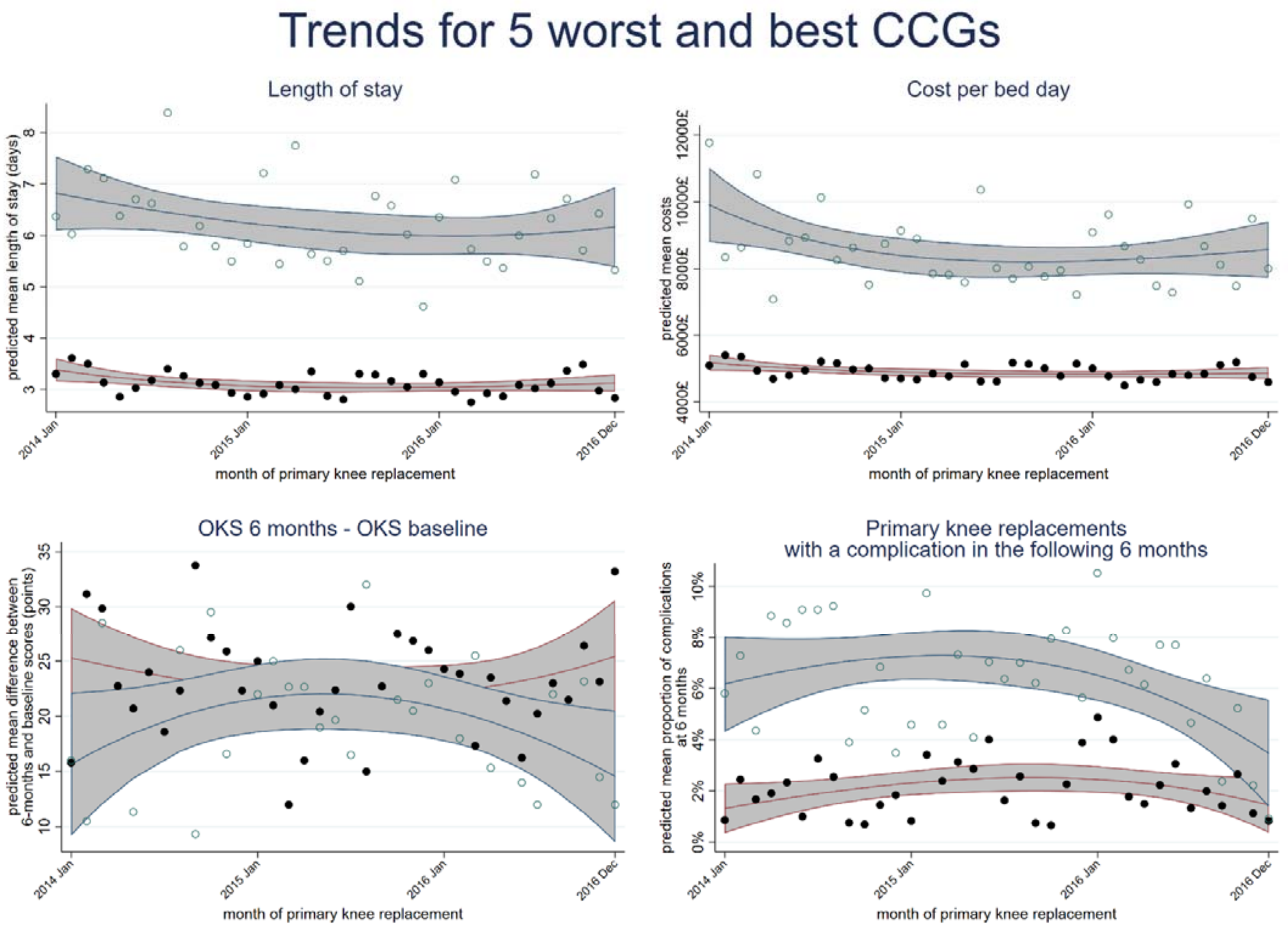

Total knee replacement, TKR; unicompartmental knee replacement, UKR; Oxford knee score, OKS; clinical commissioning groups, CCG

© 2019 Garriga C et al. *JAMA Network Open.*
